# Fruit and Seed Anatomy of *Chenopodium* and Related Genera (Chenopodioideae, Chenopodiaceae/Amaranthaceae): Implications for Evolution and Taxonomy

**DOI:** 10.1371/journal.pone.0061906

**Published:** 2013-04-23

**Authors:** Alexander P. Sukhorukov, Mingli Zhang

**Affiliations:** 1 Key Laboratory of Biogeography and Bioresource in Arid Land, Xinjiang Institute of Ecology and Geography, Chinese Academy of Sciences, Urumqi, Xinjiang, China; 2 Department of Higher Plants, Biological Faculty, Moscow Lomonosov State University, Moscow, Russia; 3 Institute of Botany, Chinese Academy of Sciences, Beijing, China; University College London, United Kingdom

## Abstract

A comparative carpological study of 96 species of all clades formerly considered as the tribe Chenopodieae has been conducted for the first time. The results show important differences in the anatomical structure of the pericarp and seed coat between representatives of terminal clades including *Chenopodium* s.str.+*Chenopodiastrum* and the recently recognized genera *Blitum*, *Oxybasis* and *Dysphania*. Within *Chenopodium* the most significant changes in fruit and seed structure are found in members of *C*. sect. *Skottsbergia*. The genera *Rhagodia* and *Einadia* differ insignificantly from *Chenopodium*. The evolution of heterospermy in *Chenopodium* is discussed. Almost all representatives of the tribe *Dysphanieae* are clearly separated from other Chenopodioideae on the basis of a diverse set of characteristics, including the small dimensions of the fruits (especially in Australian taxa), their subglobose shape (excl. *Teloxys* and *Suckleya*), and peculiarities of the pericarp indumentum. The set of fruit and seed characters evolved within the subfamily Chenopodioideae is described. A recent phylogenetic hypothesis is employed to examine the evolution of three (out of a total of 21) characters, namely seed color, testa-cell protoplast characteristics and embryo orientation.

## Introduction

The genus *Chenopodium* L. comprises at least 150 annual or perennial species distributed worldwide [1]. They are easily recognised due to the presence of flat petiolate leaves and flowers arranged in dense thyrsoid synflorescences usually called glomerules. However, *Chenopodium* was one of the most taxonomically difficult representatives of the family Chenopodiaceae. Many segregated genera were described in the 18^th^ and 19^th^ centuries, e.g. *Blitum* [2], *Morocarpus* [3], *Dysphania* [4], *Anserina* [5], *Lipandra* [6], *Oxybasis* [7], etc. (a full list is provided by Scott [8]). Their generic status was often accepted in earlier accounts (e.g. [6], [9]–[13]), but later *Chenopodium* was usually broadly circumscribed (e.g., [14]–[19]), and sometimes merged with the distinct genera *Atriplex* [20] or *Cycloloma* [21]. In the last decade the taxonomy of all Chenopodioideae has been drastically revised. As proposed by Mosyakin & Clemants [22] and confirmed from molecular results [23], all glandular representatives of the former *Chenopodium* are assigned to the core genus *Dysphania* which not only comprises the Australian species [24] but has continuously expanded with new taxa from Eurasia, Africa and America [22], [25]–[27]. In the redefined circumscription *Dysphania* may eventually comprise approximately 40 globally distributed species. Besides *Dysphania* the clade Disphanieae also contains the monotypic genera *Teloxys*, in temperate Asia, and both North American *Cycloloma* [28] and *Suckleya* [1]. The newest molecular data show that *Chenopodium* is clearly paraphyletic and split into several clades [1] specified as separate genera [29] labelled *Chenopodium* s.str. (Chenopodium s.str. clade), *Oxybasis* (C. rubrum clade), *Chenopodiastrum* (C. murale clade) and *Blitum* (Anserineae = Spinacieae clade), as well as *Lipandra* (Chenopodium polyspermum clade). Some morphological characteristics support the recent taxonomy of *Chenopodium* and related genera [29]. However, the global comparison of carpological characters, especially the fruit/seed anatomy, has not been investigated before now.

Only a set of general traits are well known for *Chenopodium* and related genera. The hyaline 1–2(3)–layered pericarp without a vascular supply is common in the subfamily Chenopodiodeae [30], [31]. The druses of crystals in the pericarp cells are relatively rare [32], [33]. The mature seed coat is 1–2(3)–layered and consists of dead cells. The exotesta (often referred to simply as the testa) performs a protective function and originates from the outer cell layer of the outer ovule integument [34], [35]. It is always easily visible since it is many times thicker than the 1(2) integumental tapetum (endotegmen) layers [36]. At maturity the cells of all seed coat layers are impregnated with tannin-like substances, making the seed colour dark brown (visually black), yellow or red. Accumulation of these substances affects the protoplast size. In the mature seed it usually decreases to just a small strip near the inner periclinal wall. Rarely the cell protoplast remains clearly visible and occupies approximately half of the cell volume (*C*. *bonus-henricus*: [30]). Moreover the testa-cell outer wall often contains darker obconical inclusions of tannin-like substances, so-called “stalactites” hanging vertically or lying obliquely. Some taxa, e.g. *Chenopodium foliosum* or *C. capitatum*, do not deposit stalactites in the testa-cell outer wall [37], [38]. Accumulation of stalactites (if present) occurs during the last stage of seed maturation [39]. The embryo is peripheral, with two well-developed cotyledons usually oriented perpendicularly to the long seed axis. The perisperm is copious.

The following carpological features are used to delimit *Chenopodium* (s.l.) species: (1) degree of fusion of stylodia, which can be free or connate at the base [40]; (2) pericarp adherent or not adherent to the seed coat [41]–[43]; (3) presence of an equatorial keel on the seed; (4) ultrasculpture of the pericarp and seed coat [37], [44]–[47]. However, the carpology of *Chenopodium* and its relatives needs reinvestigation with the following aims:

1. to clarify the diversification of fruit and seed-coat covers in the clades of the former *Chenopodium* s.l. with taxonomic implications and evolutionary trends;
2. to identify the most important traits within the entire subfamily *Chenopodioideae*;
3. to reconstruct the evolution of three taxonomically important morphological traits, namely seed color, testa-cell protoplast size, and orientation of the embryo, based on recent phylogenetic analysis of *Chenopodium* s.l.

## Materials and Methods

### Origin and Preparation of the Material

In total 67 species of *Chenopodium* s.l., another 29 representatives (from the segregate genera *Einadia, Rhagodia*, *Micromonolepis*, *Monolepis*, *Scleroblitum*, *Spinacia*) and the tribe Dysphanieae (*Cycloloma*, *Dysphania*, *Teloxys*, and *Suckleya*) were investigated. For revealing heterospermy in *Chenopodium album*, *Chenopodiastrum hybridum* and *Oxybasis glauca* the branches of the plants at different periods of the fruiting stage (July, September–October) were fixed in a 70% aqueous solution of ethyl alcohol. Some of the species under study were collected by the first author in many parts of Eurasia and preserved in 70% ethyl alcohol. No specific permits were required for the described field studies. The locations are not privately-owned or protected in any way; all gathered taxa are not endangered or protected species (mostly weeds). The herbarium specimens collected by the first author are kept at BM, E, H, K, LE, MW, and W. Other material (mostly fallen fruits) was obtained from herbarium collections (with permission) and soaked in a mixture of ethyl alcohol, water and glycerine in equal proportions. All investigated material (its origin and characters of species) is listed in Appendix S1. Anatomical cross-sections were cut either by hand or with a microtome. For tissue staining the following solutions were used: 0.2% aqueous toluidine blue to stain living tissues, Sudan IV for revealing the fatty substances and Lugol’s iodine for starch. To reveal crystals sections were viewed under polarised light. Prior to scanning electron microscopy (SEM), the material was dehydrated in aqueous ethyl alcohol solutions of increasing concentration, then in alcohol-acetone solutions and pure acetone. SEM observations were made with a JSM–6380 (JEOL Ltd., Japan) at 15 kV after critical-point drying and sputtercoating with gold-palladium. Non-dehydrated dry fruits were also used for SEM viewing for a comparison of pericarp structure. Carpological terms used are according to Werker [48].

### Character-state Reconstruction

Maximum parsimony (MP) mapping options were employed as implemented in Mesquite [49]. The TreeBase submission S12369 was used as source of both plastid and ITS BI topologies [1]. Characters were scored as multistate and non-additive.

## Results

### Pericarp

All members of the former *Chenopodium* species investigated correspond with each other in the topology of fruit and seed covers. The *pericarp* of mature fruits is always dry and often uncoloured, and it consists of one or several, rarely multiple, undifferentiated layers of parenchymatous cells. These sometimes have dark contents in some species, especially in core *Chenopodium* (*C. strictum*, *C. vulvaria*, etc.) as well as in *Oxybasis urbica*. The innermost pericarp layer, if present, often consists of thick-walled parenchymatous cells. Druses of crystals are relatively rare and are deposited in the subepidermal cell layers. In many species of core *Chenopodium* (*C. atripliciforme*, *C. strictum*, *C. nevadense*, *C. pratericola*, etc.) abundant starch grains are found.

**Figure 1:**
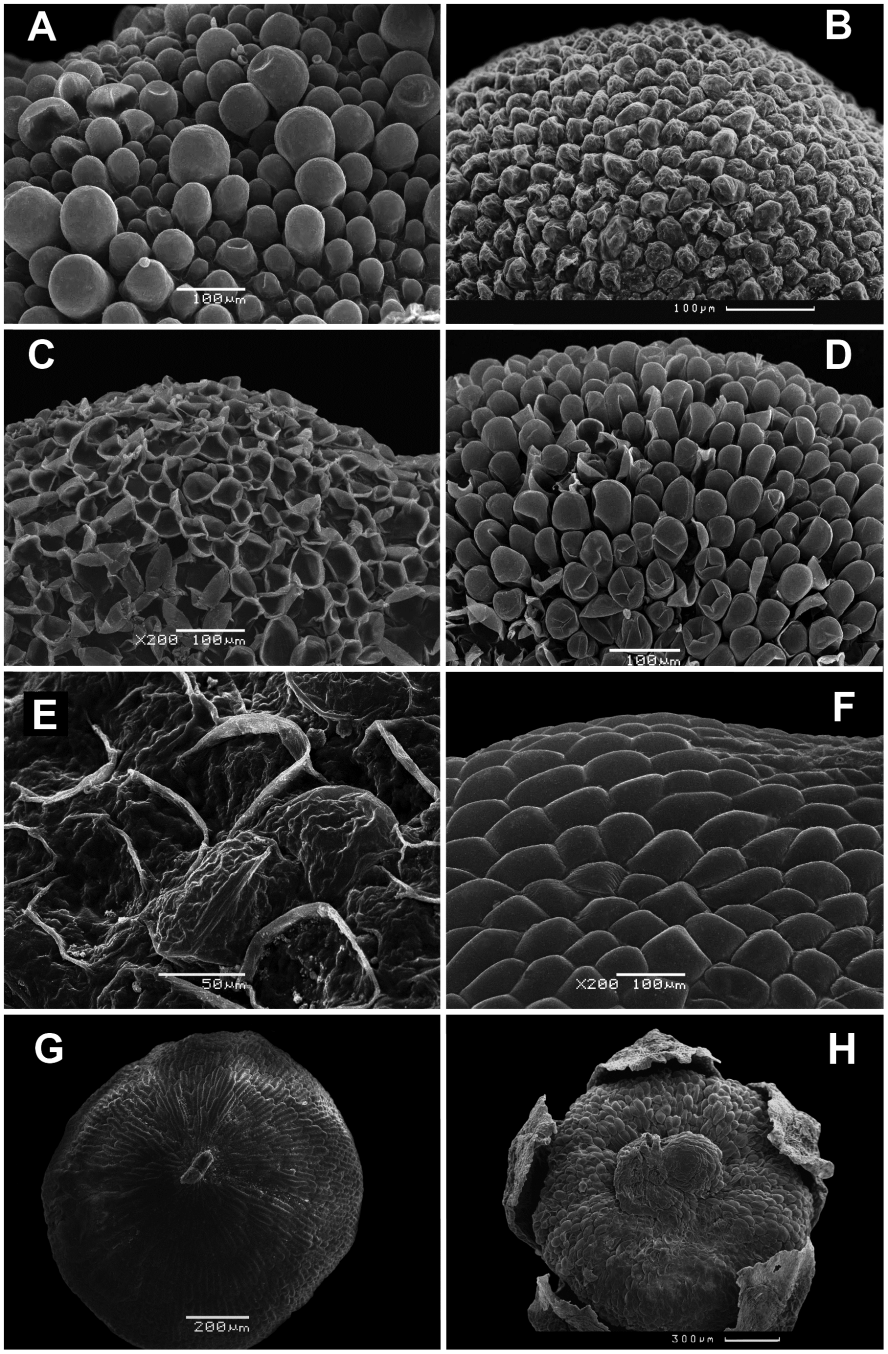
Scanning micrographs of the fruit surface. (A) Papillae on pericarp surface of *Chenopodium giganteum* (200x); (B) Papillae on pericarp surface of *Chenopodium nevadense* (250x); (C) Papillae on pericarp of dry (not soaked) fruits of *Oxybasis urbica* (200x); (D) Papillae on pericarp of *O. urbica* after standart sample preparation (200x); (E) Tiny papillae on pericarp of *Chenopodiastrum hybridum* (500x); (F) Mamillate pericarp ultrasculpture of *Blitum californicum* (200x); (G) Striate pericarp ultrasculpture of *Oxybasis glauca* (50x); (H) Fruit of *Chenopodium nesodendron* enclosed by perianth. Stylodia fallen off; swelling is seen at the apex of the fruit (50x).

Pericarp thickness in most of the species does not exceed 40–60 µm, but it can vary in many taxa due to the presence of papillae in the outer or single pericarp layer. Such protuberances of diverse (mostly cycindrical) shape up to 100–120 µm tall are common in the representatives of the core *Chenopodium* (Fig. 1A, B). In mature fruits the papillous cells often fail to maintain turgidity thereby appearing crater-like, and thus the dry fruits differ from soaked or non-abscissed ones in their surface (Fig. 1C). The cells of dry fruits of many species regain turgor pressure after soaking in water or in a glycerine-water-alcohol mixture (Fig. 1D). Only *Chenopodiastrum hybridum, C. badachscanicum, C. simplex* and *Chenopodium fasciculosum* (not involved in the molecular analysis but carpologically very close to the *Chenopodiastrum hybridum* group) fail to regain the shape of the minute papillae after soaking; the papillae are visible in immature fruits only (Fig. 1E). For this reason the crater-like pericarp surface should not be considered as a relevant taxonomic trait. The species of the former *Chenopodium* subgen. *Blitum* classified now under *Blitum* (e.g. *B. virgatum, B. capitatum*, *B. bonus-henricus*, *B. californicum*) and *Oxybasis* (*O. chenopodioides*, *C. glauca*, *C. rubra*), as well as *Lipandra polysperma*, lack papillae on the pericarp surface, which can be described either as mamillate (Fig. 1F) or reticulate/striate (Fig. 1G).

**Figure 2:**
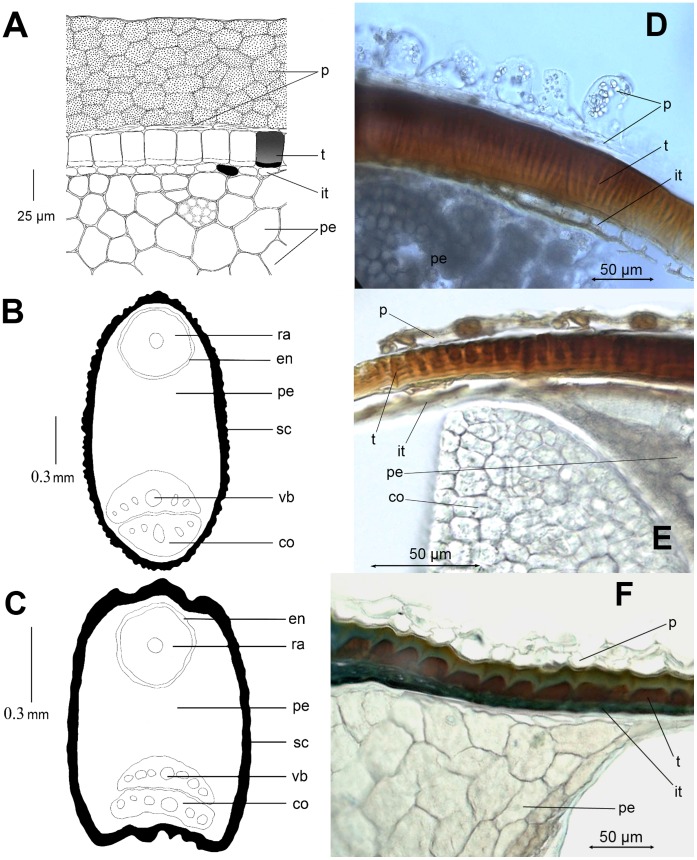
Fruit structure in cross-sections. (A) Cross-section in the central part of the fruit of *Chenopodium nesodendron*; (B) Common seed outline on example of *Blitum californicum* with alveolate testa; (C) Seed shape of *Blitum virgatum*; (D) Cross-section of fruit and seed of *Chenopodium album*. Starch grains are visible in the pericarp cells; testa cells with stalactites; (E) Cross-section of fruit and seed of *Oxybasis chenopodioides*; (F) Cross-section of fruit and seed of *Blitum californicum*. The protoplast in the cells of testa is easily visible. Abbreviations: p – pericarp; t – testa; it – integumental tapetum (tegmen); pe – perisperm; co – cotyledon; ra – radicle; sc – seed coat (testa+integumental tapetum); vb – vascular bundles in cotyledon; en – small strip of endosperm around the embryo radicle.

The thinnest pericarp (only 5–20(25) µm) consisting of one or two equal layers is found in *Blitum* (*B*. *capitatum*, *B. virgatum*, *B. petiolare* and *B. litvinovii*). The multi-layered and relatively thick pericarp is known only in a few representatives from different lineages. *Oxybasis macrosperma* possesses a multilayered pericarp varying from 50 to 130 µm on the same fruit. A robust (at least in the marginal part) and rough pericarp more than 100 µm thick forming longitudinal furrows and ribs and consisting of 5–10 (or more) layers is peculiar to *Chenopodium* sect. *Skottsbergia* (*C*. *nesodendron*, *C. sanctae-clarae*, *C. crusoeanum*). The pericarp of these three species is especially thick (up to 600 µm) at the apex of the fruit near the column due to a drastic increase in the number and thickness of the layers which form swellings (Fig. 1H; Fig. 2A).

### Pericarp and Seed Coat Adherence

The pericarp is not fused with the seed coat but affixed to it one of the following ways:

1. The pericarp is easily detached from the seed coat allowing the seed to be visible. Together with fruits, naked seeds are present in herbarium specimens of North American members of core *Chenopodium* (*C. atrovirens*, *C. boscianum*, *C. pratericola*, *C*. *nevadense*, *C. standleyanum*, *C. subglabrum*) and *Oxybasis* (*O. rubra*, *O. glauca*), as well as in *Chenopodiastrum simplex*. In cross-sections the outer pericarp layer is detached from subepidermal layers in some areas with air cavities mostly up to 100–200 µm; the entire pericarp does not tightly adhere to the seed coat;
2. The pericarp is persistent but can be readily scraped off the seed. Normally the pericarp adheres to the seed coat more or less tightly, and the cavities between pericarp and seed are present only in small areas. This type is common in many members of core *Chenopodium*;
3. The pericarp adheres to the seed coat and is hard to remove completely from the seed. It is characteristic of two members of *Chenopodiastrum* (*C. hybridum*, *C. murale*) as well as for *C. badachschanicum* and *Chenopodium fasciculosum*, and also for *Blitum* (*B. capitatum*, *B. virgatum*, and related taxa of the former *Chenopodium* sect. *Blitum*). Rarely it is evolved within the core *Chenopodium* (North American *C. pallescens*).

### Seed Outlines

*Chenopodium* s.l. species have slightly depressed seeds with ovoid outlines in cross-section. The length (here simplified as diameter)/thickness ratio is 1.5–2∶1 (Fig. 2B). Seeds of some core *Chenopodium* and two representatives of *Chenopodiastrum* (*C. murale*, *C. coronopus*), as well as *Chenopodium fasciculosum* and *Chenopodium gubanovii* possess a median keel forming a sharply acute seed-margin outline. The Eurasian *Blitum virgatum* complex (*B. virgatum* s.str., *B. petiolare*, *B. litvinovii*) is clearly distinct from other *Blitum* species through the presence of the marginal groove and two obscure keels (Fig. 2C). The seed outlines correspond with those of the fruit.

### Seed Colour

The black seeds are usual for the members of core *Chenopodium*. Together with the black seeds, yellow (or yellow-brownish) ones can be present in the same plant (a case of evident heterospermy, found in *C. album* and *C. pamiricum*). The (dark) red seeds are common in representatives of *Blitum* and *Oxybasis* (except *O. urbica* with black seeds).

### Seed Coat

The crustaceous consistency of the seed coat is defined by its testa. In cross section the testa can be smooth, undulate or clearly alveolate. Sometimes the outline of the testa is undulate or pitted only in the marginal part of the seed, and smooth in the central part, as in *C. gubanovii*. The depth of the crater-like recessions (often called combs) which account for the pitted seed structure can be insignificant (±5 µm) or can reach 20–25 µm (especially in *Chenopodiastrum hybridum* and relatives).

In the majority of *Chenopodium* and relatives the testa thickness varies from (15)20 to 50 µm (Fig. 2D). Such variation results from the presence of heterospermy (at least in taxa with comprehensive statistical samplings) or thickening of the seed coat in some parts of the seed, predominantly at its margins (±10 µm). Despite the fact that the seeds appear to be red or black, all layers are dark brown in cross section. The seeds with a thin (5–15 µm) yellow testa dominate only in *C. pamiricum*, *C. pallidicaule* and *C. quinoa*, and are unusual for all lineages of earlier *Chenopodium*. On the contrary the seed coat of *Chenopodiastrum hybridum* and relatives is ordinarily much harder and varies in thickness from 35–50 to 100–120(150) µm according to the heteromorphic seed type. Many North American core *Chenopodium* (*C. berlandieri*, *C*. *boscianum*, *C. hians*, *C. incanum*, *C. subglabrum*) are distinguished by thickening of the testa layer (45–100 µm) that could be explained as response to extreme arid conditions.

The testa cells of the mature seed usually have small strip-like protoplasts (Fig. 2D, 2E). The only exceptions are *Blitum bonus-henricus* and *B. californicum* with easily visible and uncompressed protoplasts in the testa cells (Fig. 2F). In many species the outer wall of the seed testa is impregnated with tannin-like substances (stalactites). The most common orientation of the stalactites is vertical (radial). *Chenopodiastrum hybridum* and relatives (*C. badachschanicum*, *C. simplex* and *Chenopodium fasciculosum*) and are characterised by obliquely hanging stalactites. The thin testa of yellow seeds of *C. quinoa*, *C*. *pamiricum* lacks stalactites. The same applies to the testa of *B*. *bonus-henricus*, *B. californicum*, *B. virgatum*, *B. capitatum*, *B. petiolare* and *B. litvinovii*.

An anatomical description of each investigated species is given in Appendix S1.

## Discussion

Recent molecular studies show that *Chenopodium* s.l. is non-monophyletic and consists of six independent lineages [1], which generally correspond to several former *Chenopodium*-segregated genera and the newly described genus *Chenopodiastrum* [29]. The carpology of all of these taxa require detailed analysis.

### Heterospermy: Conclusions

In taxonomic accounts and even in specialised carpological articles the reproductive diaspores of *Chenopodium* are considered to be uniform [17], [35], [50]–[55]. However, some results have shown the presence of heterospermy, especially in *C. album*, as one of the most widely distributed and taxonomically complex species, but these data often appear to be inconsistent.

Baar [56] made the first attempt to describe seed heterogeneity in *C. album*. He observed the presence of both black and brownish seeds within an individual. This work is seldom cited because of difficulties in visualising the second seed type. Only Baygozina et al. [57] indicated later the evident heterospermy in *C. album* but without any explanation. On the contrary some authors [58]–[62] postulated the existence of cryptic heterospermy manifested by the presence of black seeds of various sizes and of their capability for rapid or delayed germination. The origin of cryptic seed heterogeneity is connected with day length: during long-day periods the plants produce predominantly seeds with a robust testa [63]. Our own investigations show that in the summer (long days) the seeds of *C. album* developing from terminal and lateral flowers have a thick (30–50 µm) testa. By contrast the autumn seeds (chiefly from lateral flowers evolving after the terminal flowers: [64], [65]) have a thinner (17–25 µm) testa. Moreover, a recent study of seed heterogeneity in *C. album* confirmed that, as well as black seeds, there are yellow-brownish ones formed under unfavourable environmental conditions, e.g. salinity stress [66]. Such yellow-brownish seeds are usually absent from plants growing in ruderal sites. Thus *C. album* demonstrates trispermy with a prevalence of cryptic heterospermy, but the possibility of presence of all three seed types on the same plant requires further investigation.

The representatives of the core *Chenopodium* having all or most seeds with a thin yellow testa are *C. quinoa* and *C. pallidicaule* (both of South American origin), and the Central Asian *C. pamiricum*. However, the yellow seeds of *C. quinoa*, which is regarded as an important crop in the tropics, are the result of selective breeding and were initially dark [67]. This opinion is supported by the presence of papillae in the pericarp enveloping the yellow seed. No other case in the entire Chenopodioideae is known in which the pericarp of fruits with a thin yellow testa possesses prominent papillae on its surface (Sukhorukov, unpubl.). All examined specimens of *C. pamiricum* produce dark seeds in extremely limited numbers. They are rounded, keeled, and with a 17–25 µm thick testa containing stalactites. The yellow seeds, on the contrary, are oblong and lack a keel; the testa is 5–8 µm thick and lacks stalactites. Heterocarpy is also present: the pericarp of fruit containing dark seeds has papillae, in contrast to the pericarp of fruit with yellow seeds.

Another taxon with hidden heterospermy that has been examined is *Chenopodiastrum hybridum*. Careful examination of the seed produced by *C. hybridum* shows that there is no connection between testa thickness and seed diameter. As in *Chenopodium album* seeds with a thick testa (75–110 µm) are produced in both terminal and lateral flowers when the days are long, whereas those with a thinner testa (30–50 µm) form towards the end of the growth period. The alveolate structure of the testa surface (±20–25 µm) accounts for the considerable variation in thickness in each seed type.

Representatives of *Oxybasis*, especially *O. glauca*, *O. rubra* and *O. chenopodioides*, often exhibit spatial heterospermy (cf. [68]) connected with seed position within the dichasial inflorescences. Commonly the seed embryo in the terminal fruits point vertically, while in the lateral ones the embryo is horizontally oriented. Other differences between these two seed/fruit types have not been observed. But cryptic seed heterogeneity is found in horizontally oriented seeds of *O. glauca* with testa thicknesses of 10–15 µm and 17–25 µm respectively (Sukhorukov [69], sub *Chenopodium glaucum*). Structural heterocarpy has not been observed in *Chenopodium album*, *Oxybasis glauca* or *Chenopodiastrum hybridum*.

### Carpology of Other Taxa Formerly Considered Members of the Tribe Chenopodieae

#### Tribe Dysphanieae

The most indicative trait of this group is presence of glandular hairs, glands and (or) simple hairs on the stem, leaves or perianth which often impart an aromatic smell to the whole plant.

##### Dysphania R.Br. (incl. Roubieva Moq.)

Cosmopolitan genus including representatives which either have a restricted range or are common weeds in (sub)tropical regions of the Old and New Worlds [46], [70]. Although the floral histogenesis of some *Dysphania* species is similar to that of *Chenopodium* [71]–[73], the characteristics of the fruit and seed covers are distinct and thus taxonomically reliable. The pericarp is thin, 3–10(15) µm, 1–2-layered, and adheres tightly to the seed coat (except *D. tomentosa* with easily ruptured pericarp). The testa is only 7–15(20) µm thick.

**Figure 3:**
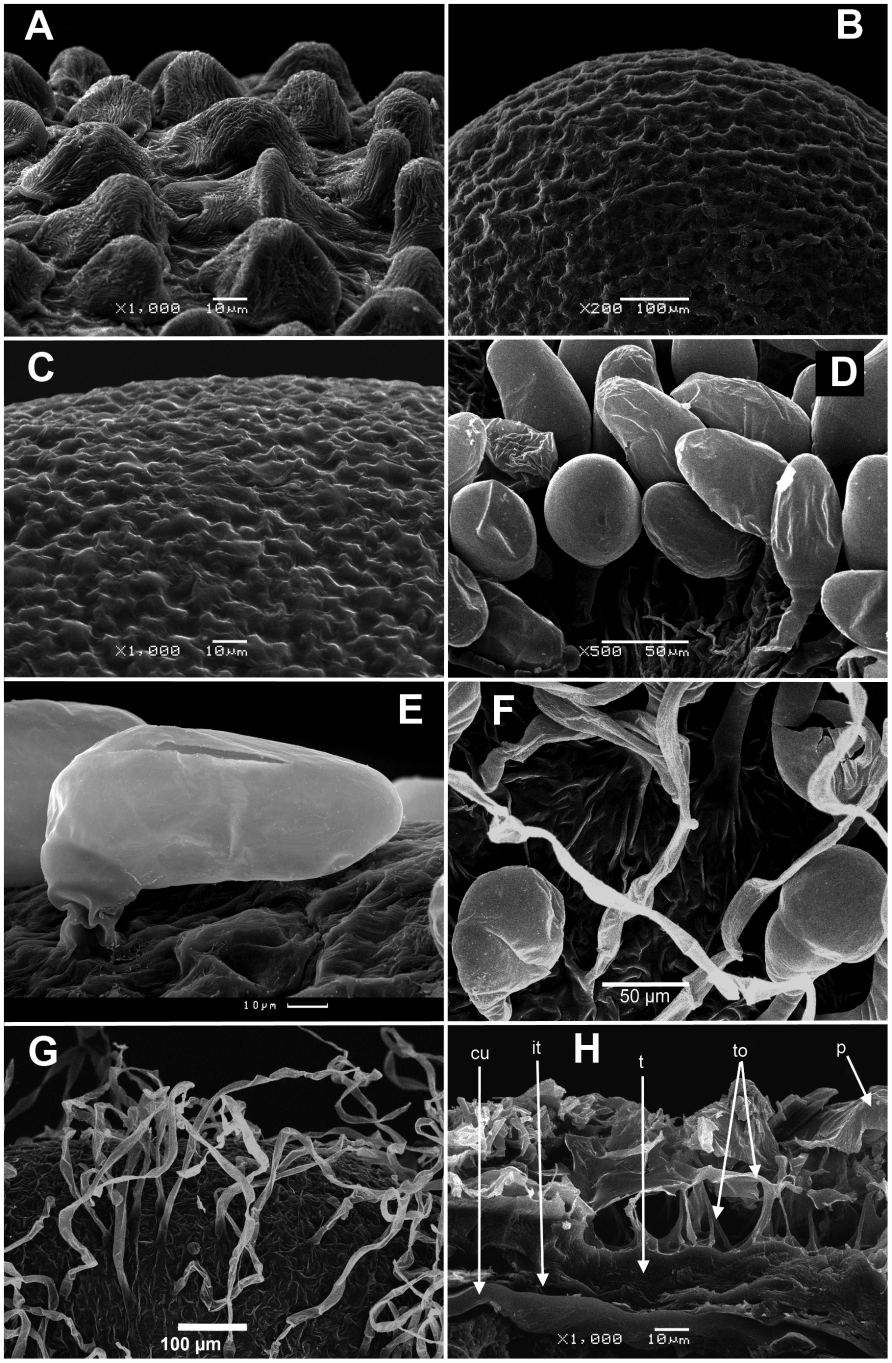
Scan micrographs of the fruits. (A) Papillae on pericarp surface of *Dysphania procera* (1000x); (B) Pericarp surface of *D. congolana* (200x); (C) Tiny papillae on pericarp surface of *D. pseudomultiflora* (1000x); (D) Glandular trichomes on the pericarp surface of *D. chilensis* (500x); (E) Glandular trichomes on the pericarp surface of *D. multifida* (1000x); (F) Glandular trichomes and central parts of curved simple hairs on the pericarp surface of *Cycloloma atriplicifolium* (500x); (G) Simple curved hairs on the pericarp surface of *C. atriplicifolium* (200x); (H) Cross-section of the pericarp and seed coat of *Blitum atriplicinum* (1000x) showing acicular outhgrowths of the testa cells. Abbreviations: p – pericarp; t – testa; to – testa outgrowths; it – integumental tapetum; cu – cuticle between integumental tapetum and perisperm.

The fruit and seed-structure characters support the division of the genus in a recent revision into several geographically localised groups:

1. Australian taxa with minute (0.3–0.65 mm) fruits that are apparently not found in any species of Chenopodioideae. Pericarp smooth with reticulate ultrasculpture or with tiny papillae. Seeds very different in shape, globose, oblong with groove, keeled or not [46], as a rule with a vertical and almost straight (not curved) embryo [71], [74]. This orientation of the seed embryo is not found in the subfam. Chenopodioideae;
2. Eurasian, African+one North American (*D*. *graveolens*) taxa. Fruits 0.6–0.9 mm in diameter, subglobose, with length/thickness ratio 1.2–1.3∶1. The pericarp with small conical or cylindrical papillae (Fig. 3A) is an additional character for delimitation of the species (Sukhorukov, in prep.), rarely (almost) lacking pericarp outgrowths (*D. congolana*: Fig. 3B, *D* *. pseudomultiflora*: Fig. 3C). Seeds with horizontal and peripheral curved embryo;
3. American species with larger (0.7–1.5 mm) subglobose or broadly ovoid fruits (length/thickness ratio 1.3–1.4∶1) having prominent glandular (vesicular) hairs with a few-celled stalk (*D. ambrosioides*, *D. anthelmintica*, *D. chilensis*: Fig. 3D, *D* *. multifida*: Fig. 3E, *D* *. bonariensis* and other American species (see also [75]) that are not observed in the pericarp of any other lineage, in contrast to earlier data [76]. The seeds have a horizontal or rarely both horizontal and vertical (in *D*. sect. *Adenois*, after Clemants & Mosyakin [55]) peripheral curved embryo. *D. multifida* and *D. bonariensis*, previously included in *Roubieva* (as *R. multifida* and *R. bonariensis* respectively), differ from other taxa mainly in the balustriform and hardened perianth with segments that are fused at its apex.

##### Teloxys Moq

one non-aromatic species, *T. aristata,* in Central Asia and as an ephemerophyte in many parts of Europe and North America. It is easily recognised by having several (sub)sessile leaves often folded on the ventral side, and acicular branches. Rarely, especially on moist substrates, the plants fail to develop acuminate apices (Iljin in herb. LE), and for this reason the differences between *Teloxys* and *Dysphania* were not previously clear [22], [77]. The most important carpological trait of *Teloxys* is the flattened shape of the fruits (and seeds) with a length/thickness ratio of 2∶1. Other characteristics of *Teloxys* (lack of papillae on the pericarp surface, prominent seed keel) are not shared with all Eurasian and both South/North American *Dysphania*.

##### Cycloloma Moq

one species, *C. atriplicifolium* in North America, known also as an alien in Australia, Western and Central Europe [78]. Morphologically the genus is characterised by three stylodia and a persistent perianth with a horizontally oriented wing-like appendage near its middle. This resemblance in the perianth character to many *Camphorosmioideae* was the reason for transferring *Cycloloma* to this subfamily [79]. However, the hard seed testa is atypical for this group although general within the Chenopodioideae. Other notable traits of this genus not already mentioned include: (1) the perianth in its basal part adheres to the thin (1–2–layered) pericarp that readily detaches from the seed coat, and (2) the pericarp is covered with trichomes of two types: glandular hairs with a large terminal cell (Fig. 3F) as in the American *Dysphania*, and long curved simple hairs (Fig. 3G). The second indumentum type is not mentioned by previous authors for any examined representatives of the Chenopodioideae (Sukhorukov, unpubl.).

##### Suckleya A. Gray

The systematic position of this North American genus was variable up to now [80], [81], and only recently was it included in the tribe Dysphanieae [1], [28]. Carpologically the genus is distinguished from other members by to the large, compressed fruits and the yellow colour of the seeds. However, it resembles other Dysphanieae in some of characters, namely the very thin, 1-layered and appressed pericarp with small protuberances of the outer cell walls, and the small testa lacking stalactites.

### The Remaining Genera Examined Carpologically

*Rhagodia* R. Br. and *Einadia* Raf. The fruits of *Rhagodia* are heterocarpic with diverse (red, yellow and dark) pigmentation of the pericarp [82], [83]. The pericarp, at least of the red and white fruits, also varies in thickness. The so-called dark fruits have in fact a colourless pericarp, and the colour is imparted to the fruit by the black seed that is visible through the thinner (130–150 µm) pericarp comprising 5–7 layers of non-inflated cells. In *Einadia nutans* some of the heterocarpic fruits have a thin, 1–2–layered, white pericarp which make them indistinguishable from the fruits and seeds of the vast majority of core *Chenopodium*. The close relationship of *Rhagodia* and *Einadia* to *Chenopodium* s.str. proposed by Diels and Pritzel [84] or Dinan et al. [85] can be confirmed by the following fruit and seed characteristics: (1) some of the fruits of *Rhagodia* and *Einadia* have a colourless (white) pericarp as in the core *Chenopodium* [86], while a dark-coloured (but thin) pericarp not infrequently appears in some *Chenopodium;* (2) presence of a multilayered pericarp in *C*. sect. *Skottsbergia*; (3) presence of (visually) black seeds. However, Scott [87] separated the subtribus *Rhagodiinae* within the former tribe Chenopodieae s.l. with the incorporation of three genera (*Rhagodia*, *Einadia* and *Holmbergia*) having berry-like fruits. The structure of such fruits cannot be regarded as equivalent (for more details see [28]), and both molecular and carpological data support *Rhagodiinae* being a heterogeneous group: Australian *Rhagodia* and *Einadia* are nested within the core Chenopodium lineage with the new nomenclatural combinations [1], and the South American *Holmbergia* belongs to the Archiatriplex clade [28], [88].

*Monolepis* Schrad. had included three annual species with disjunct distribution: *M. asiatica* in Arctic Siberia and both *M. spathulata* and *M. nuttalliana* in temperate America [53]. They were distinguished by the drastic reduction in the number of perianth segments (up to 1–2) and lateral flowers in the cymes [38], [89]. Recently all members of *Monolepis* have been transferred to *Blitum* by Fuentes-Bazan et al. [29]. The pericarp of all representatives is one- or few-layered and readily removed. The seeds are small, especially in *B. spathulatum*, with a very thin testa (7–12 µm). In the arctic species *B. asiaticum* the pericarp and seed coat are very thin and so the hypothesis concerning a hardened pericarp or seed coat in the Chenopodiaceae clade providing additional embryo protection [90] is not confirmed in the present study.

Other characteristics are distinct for each species. *Blitum asiaticum* possesses keeled seeds 0.9–1 mm long with an undulate testa lacking stalactites. In *B. spathulatum* the seeds do not have a keel and the testa contains stalactites. *Blitum nuttallianum* has a peculiar seed testa with slender hair-like tangled outgrowths.

*Micromonolepis* Ulbr. contains one west-American, short-lived, annual endemic *M. pusilla*. The genus described by Ulbrich [16] is currently accepted [28], [91]. It is distinguished by fleshy leaves and a dichotomous-like branching pattern (Torrey [92], sub *Monolepis pusilla*). Carpologically *M. pusilla* does not differ significantly from *Monolepis spathulata*.

*Scleroblitum* Ulbr. One species *S. atriplicinum* of Australian origin is now included within *Blitum*. As in American *B. nuttallianum*, the seed testa cells develop hair-like projections which adhere tightly to the 1–2(3)–layered pericarp (Fig. 3H).

*Spinacia* L. Three species in Eurasia, and one of them *S. oleracea* is widely cultivated as a vegetable. The most remarkable traits of *Spinacia* are the unisexual flowers and the bract-like cover of the female flowers formed by accrescent and fused perianth segments [93], [94]. The pericarp is 1–layered and very thin (ca. 5 µm), lacks papillae, and tightly adheres to the seed coat. The testa thin, yellow, and lacks stalactites.

### Set of the Fruit/Seed Characters in Chenopodioideae

A set of phylogenetically important characters is given below.

1. Stylodia: 1– free; 2– concrescent through most of the column; 3– single stylodium;
2. Average fruit diameter: 1 – 0.3–0.6 mm; 2 – 0.7–1.6 mm; 3 – 1.6 to 3 mm; 4 – 3–10 mm;
3. Fruit length/thickness ratio: 1 – almost equal (fruits subglobose); 2 – length significantly greater than thickness (fruits flattened);
4. Pericarp adherence to the seed coat: 1 – easily detached; 2 – readily scraped off the seed; 3– pericarp adherent to the seed coat;
5. Pericarp detachments: 1 – not visible to the naked eye (anatomically visible); 2 – detachments ear-like, present in the upper part of the fruit; 3 – detachments can develop in different parts of the fruit;
6. Pericarp consistency: 1 – dry; 2 – tendency to be fleshy and coloured;
7. Pericarp outlines: 1 – not rough (not foveolate); 2 – clearly foveolate;
8. Pericarp layers: 1 – 1–2(3) layers; 2 – more than 3 layers (at least in some fruits);
9. Outer cell wall of the outer pericarp layer: 1 – papillate (at least in the majority of fruits); 2 – not papillate (smooth) or with mamillae only (papillae can sometimes be present in a particular part of the fruit); 3 – with vesicular trichomes; 4 – with vesicular trichomes and simple hairs; 5 – with stellate hairs;
10. Pericarp topography: 1 – undifferentiated (parenchymatous or rarely parenchymatous sclerenchyma); 2 – differentiated into parenchyma (sometimes sclerified parenchyma) and sclerenchyma (at least in some fruits, if heterocarpic);
11. Exocarp: 1 – one-layered only; 2–2–5-layered;
12. Seed colour: 1 – black or black and brownish (if heterospermous); 2 – red or reddish-black; 3 – brown (or yellow-brown) only; 4 – unpigmented; 5 – red and brown (heterospermy);
13. Keel on the seed: 1 – no keel or slightly keel; 2 – with one or two prominent keels;
14. Differentiation of the seed coat: 1 – clearly differentiated into thick testa and thin tegmen; 2 – not clearly differentiated, all layers thin or (almost) equal; 3– both types (heterospermy);

1. Sculpture of the testa: 1 – not or slightly undulate; 2– alveolate;
2. Testa thickness: 1 – up to 20(25) µm (at least in the majority of the fruits); 2 – from 20 to 50(60) µm; 3 – both types 1 and 2 due to obvious heterospermy; 4 – more than 50 µm;
3. Stalactites: 1 – vertically oriented (present in the black and red seeds); 2 – obliquely oriented; 3 – lacking prominent stalactites; 4 – both types, i.e. some seeds with and some without (heterospermy);
4. Protoplast of the testa cells: 1 – always compressed due to impregnation of the outer cell wall with tannins; 2 – easily visible.
5. Embryo orientation: 1 – horizontal; 2 – both vertical and horizontal within an individual; 3– vertical;
6. Embryo curvature: 1 – annular (curved) or horseshoe-shaped; 2 – straight or slightly bent;
7. Hair-like outgrowths from the testa: 1 – absent; 2 – present.

Three characters of major taxonomic importance (12, 18, 19) were chosen for ancestral character mapping.

All our reconstructions, however, should be treated with caution due to the hard incongruence between plastid and ITS trees [1] and the ability of monodirectional concerted evolution of rDNA copies. All reconstructions show the dynamic nature of reconstructed characters.

### Character 12 (Seed Color). ITS Topology

**Figure 4:**
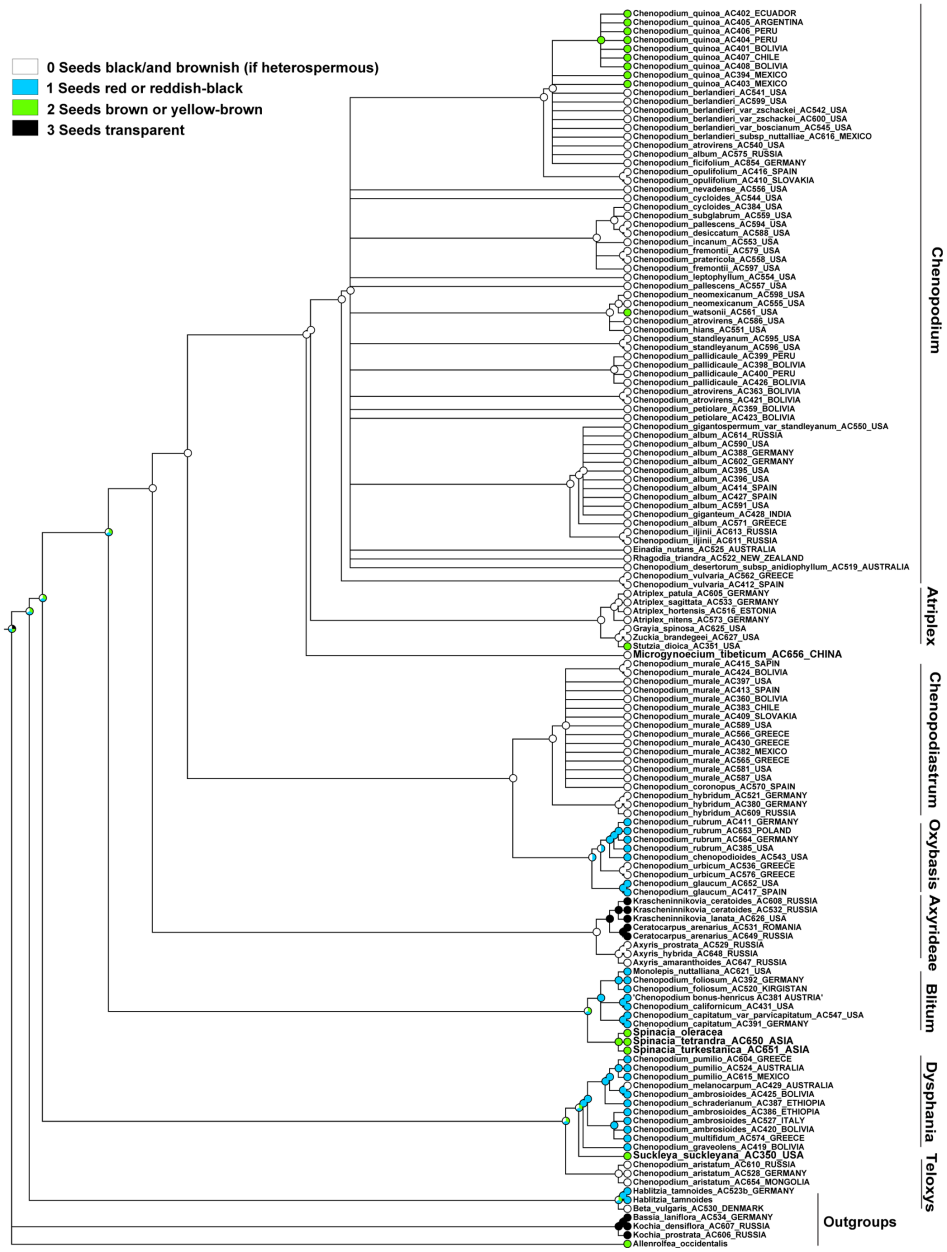
MP reconstruction of seed colour. ITS/BI topology (Fuentez-Bazan & al., 2012a).

The MP reconstruction shows that the ancestral state of the Atripliceae+Anserinae+Dysphanieae +Axyrideae clade is equivocal. It also shows that black or black/brownish seeds evolved as ancestral to all major clades after Axyrideae, red or reddish-black seeds are the ancestral state for the Oxybasis, Blitum, and Dysphania clades, and they evolved as a putative homoplasy in the small *Habilitzia-*clade (outgroup: Fig. 4). Brown seeds evolved independently many times: (some core *Chenopodium*), *Stutzia dioica*, Spinacia-clade, *Suckleya suckleyana*, *Allenrolfea occidentalis* (outgroup). Unpigmented seeds evolved as a homoplasy within *Bassia* (incl. *Kochia*) as the outgroup and within the Axyrideae clade in *Krascheninnikovia* and *Ceratocarpus*.

### Character 12 (Seed Color). Plastid Topology

**Figure 5:**
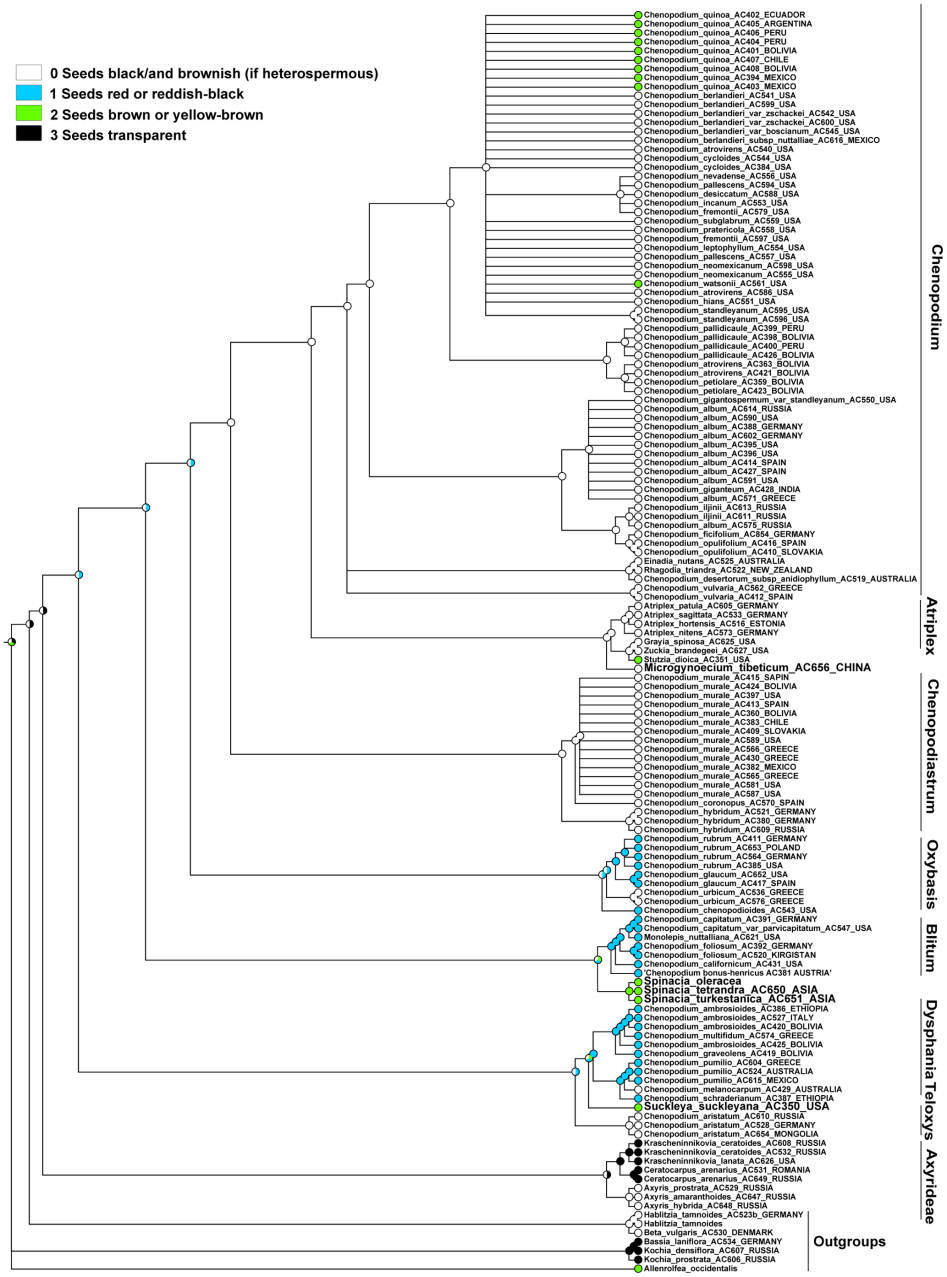
MP reconstruction of seed colour. *trnL-F*/BI topology (Fuentez-Bazan & al., 2012a).

The MP reconstruction shows that the ancestral state of the Atripliceae+Anserinae+Dysphanieae +Axyrideae clade is equivocal, but black or black/brownish seeds are ancestral to the Chenopodium+Atriplex+Microgynoecium+Chenopodiastrum-clade as well as to the clades *Chenopodium*, *Atriplex*+*Microgynoecium,* and Chenopodiastrum, and evolved as a putative homoplasy of *Oxybasis urbica* and *Dysphania melanocarpa* (Fig. 5). Red or reddish-black seeds evolved as a putative homoplasy within the *Oxybasis*, *Blitum*, and *Dysphania* clades and maybe also in all clades after Axyrideae. Brown seeds evolved as a homoplasy of *Suckleya*, *Spinaceae*, *Stuzia* and some core *Chenopodium*. Due to the presence of brown seeds in *Allenrolfea* (outgroup) we interpret all these cases of homoplasy as reversals. Unpigmented seeds seem to be homoplastic in the *Krascheninnikovia*+*Ceratocarpus-*subclade (Axyrideae clade) and the Bassia incl. Kochia clade (outgroups).

### Character 18 (Protoplast of the Testa Cells). ITS and Plastid Topologies

**Figure 6:**
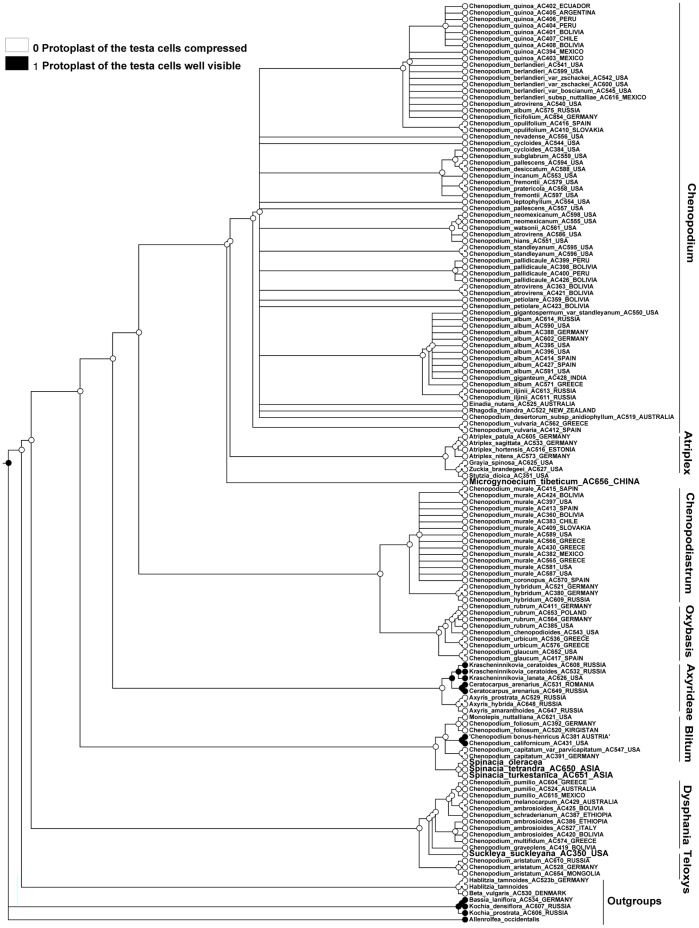
MP reconstruction of the protoplast size of the testa cells. ITS/BI topology (Fuentez-Bazan & al., 2012a).

**Figure 7:**
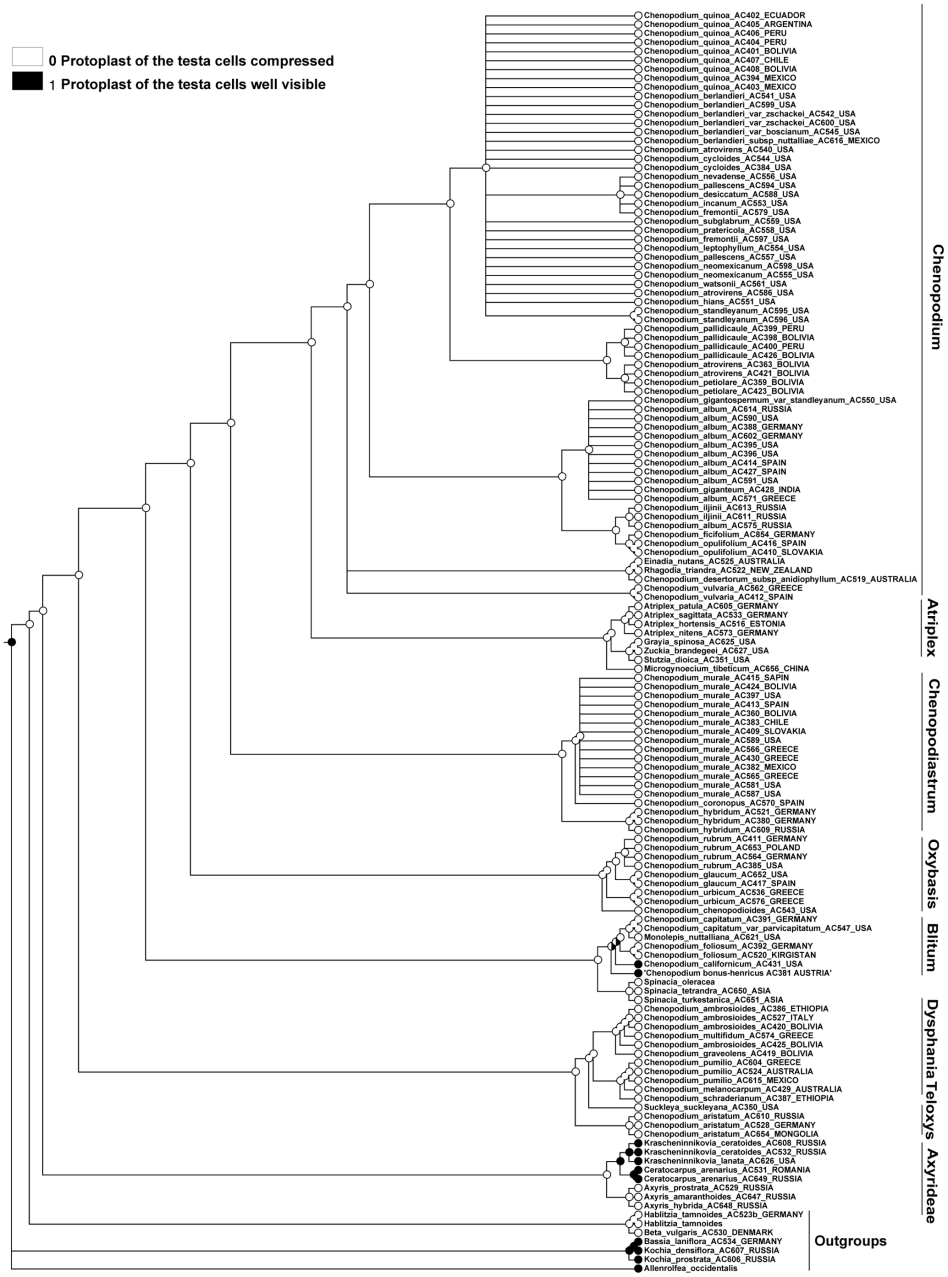
MP reconstruction of the protoplast size of the testa cells. *trnL-F*/BI topology (Fuentez-Bazan & al., 2012a).

MP reconstructions (both ITS and plastid) show that the easily visible protoplast of the testa cells evolved as a homoplasy within outgroups (e.g. the Krascheninnikovia+Ceratocarpus-subclade) and the Blitum clade (Fig. 6, 7). For the majority of Chenopodioideae the decreasing in cell volume is connected with impregnation of the outer cell wall with tannins and (often) the emergence of the stalactite-like strengthening of the cell walls.

### Character 19 (Orientation of the Embryo). ITS Topology

**Figure 8:**
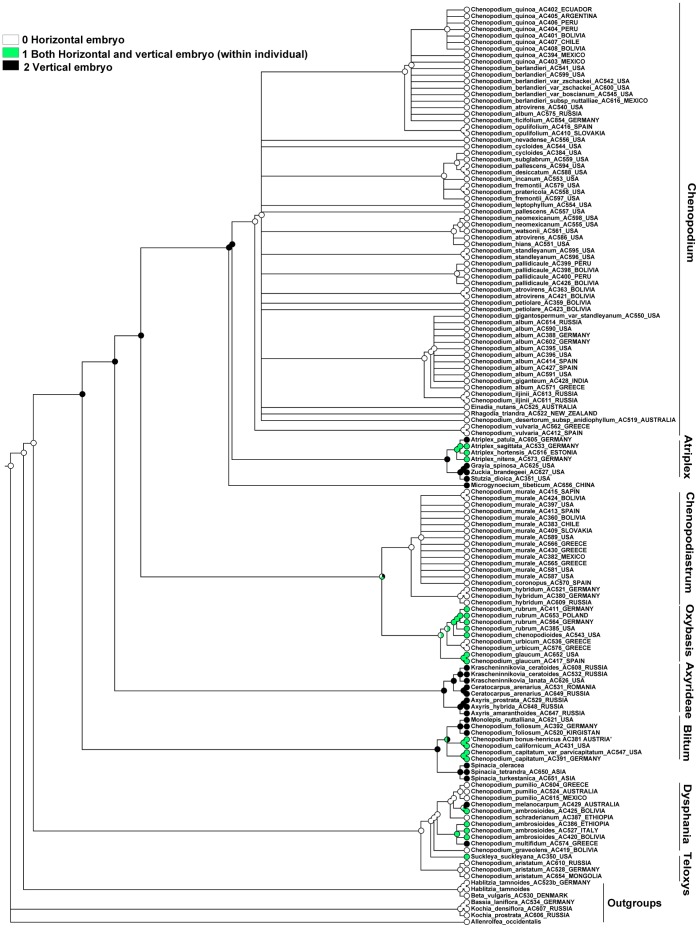
MP reconstruction of the orientation of the embryo. ITS/BI topology (Fuentez-Bazan et &., 2012a).

The MP reconstruction (Fig. 8) shows that the horizontal orientation of the embryo is ancestral for the Atripliceae+Anserinae+Dysphanieae+Axyrideae clade, but after the Dysphanieae clade this was switched to a vertical orientation, which in our reconstructions is the ancestral state for the next six deepest nodes (up to the ancestor of core *Chenopodium* that reverted back to a horizontal orientation). The character where both vertical and horizontal embryos are found within an individual evolved homoplastically in numerous branches and clades and is reconstructed as the ancestral state of the *Oxybasis rubra*+*O. glauca* subclade. The horizontal embryo also evolved as a reversal in the Dysphania-clade (at least in *D. multifida* and *D. melanocarpa*).

### Character 19 (Orientation of the Embryo). Plastid Topology

**Figure 9:**
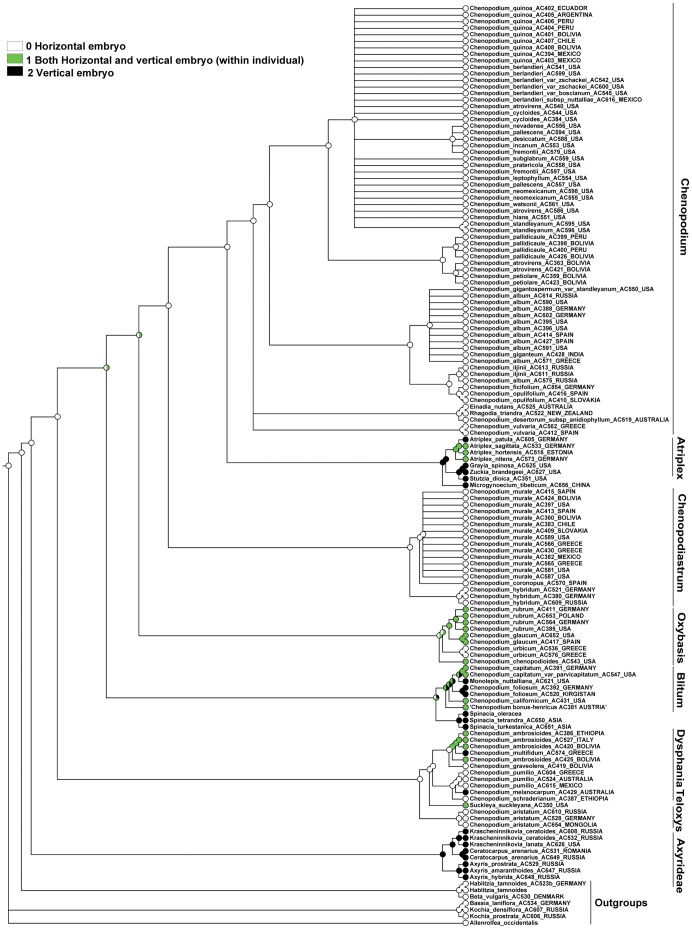
MP reconstruction of the orientation of the embryo. *trnL-F*/BI topology topology (Fuentez-Bazan & al., 2012a).

The MP reconstruction (Fig. 9) shows that the ancestor of the Atripliceae+Anserinae+Dysphanieae+Axyrideae clade, as well as those of the *Chenopodium*, *Chenopodiastrum*, *Dysphania*, and *Dysphania*+*Teloxys* clades, have a horizontal embryo, and the ancestral state of the Axyrideae, Spinaceae, and *Atriplex*+*Grayia*+*Zuckia*+*Microgynoecium* clades might have both vertical and horizontal embryos within an individual. In contrast the ancestral character state of the *Oxybasis* and *Blitum* clades is equivocal. All types of orientation evolve with some degree of homoplasy.

Generally a vertical embryo is observed in all one-seeded Caryophyllales except for some Chenopodiaceae (Sukhorukov & al., in prep.). Therefore the horizontal embryo of the family groups is an unusual derived trait.

### Does Carpology Support the Recent Reconstructions of Chenopodium Lineages based on Molecular Results?

The first molecular classification of Chenopodieae s.l. into unranked Chenopodieae I and II proposed by Kadereit et al. [28], [88] was the most consistent with the carpological findings. The group Chenopodieae I could have included taxa with visually black seeds and horizontal embryos, and the most remarkable differences are in terms of the pericarp structure. Chenopodieae II might have been distinguished by (dark) red or brownish seeds and vertical (or both vertical and horizontal) embryos. The recent division of *Chenopodium* into several independent lineages [1] makes the situation with regard to fruit/seed structure more complicated.

According to Fuentes-Bazan et al. [29] one comprehensive clade includes several different lineages of *Chenopodium* s.l. (*Chenopodium* s.str., *Chenopodiastrum*, *Lipandra*, *Oxybasis*) as well as *Atriplex* and its relatives. Taxonomically it must be called the tribe Chenopodieae sensu novo (not Atripliceae!) associated with the type *Chenopodium album* [95], and the tribe Atripliceae should therefore be considered as a synonym of Chenopodieae.

### Chenopodium s.str

The species are distinguished by green perianth segments and visually black seeds with horizontal embryo. This group seems to have specialized bladder hairs on vegetative organs and the perianth [96] in contrast to other phylogenetic lineages. Three endemics of the Juan-Fernandez Archipelago (*C. sanctae-clarae* and relatives) possess, in addition to the unusual small tree-like habit [97], other remarkable characters such as a multi-layered unpigmented pericarp and a rough, thick seed coat.

### Chenopodiastrum

All members of this group resemble the core *Chenopodium* (papillae on the pericarp surface; presence of black seeds, etc.) Within *Chenopodiastrum* the fruit/seed structure between both subclades (*C. murale* and *C. hybridum* aggregates) differs in relevant characters (fruit size, shape of papillae, testa thickness). Within the *C. hybridum* group the degree of adherence of the pericarp to the seed coat is extremely variable. The relationship of *Chenopodium fasciculosum* to *Chenopodiastrum* needs to be clarified. Almost all carpological traits of this taxon indicate a very close affinity to the *Chenopodiastrum hybridum* group except for the presence of the seed keel, which was the reason for considering *Chenopodium fasciculosum* to be closely related to the subclade *Chenopodium murale* [98].

### Lipandra

This differs from the two genera already mentioned by the pericarp lacking papillae.

### Oxybasis

Only a few representatives are involved in the molecular analysis. The close relationship between *O. rubra*, *O. macrosperma*, *C. chenopodioides* and *C. glauca* is undoubtedly supported by the carpology (but cf. Williams [99]). Carpologically the most remarkable taxon is *O. macrosperma* with its multi-layered pericarp. Besides, other representatives now called *Chenopodium gubanovii*, *C*. *antarcticum* and *C. mexicanum* can complement this genus. The inclusion of *C. urbicum* in *Oxybasis* is surprising from a carpological point of view.

### Blitum

This lineage is characterised by unusual life histories (obligate perennial herbs, such as *B. bonus-henricus* and *B. californicum*, or facultative short-lived perennials in the *B. virgatum* group: Uotila [100], sub *Chenopodium foliosum* group). Carpologically *Blitum* is still the most heterogeneous group. However, both molecular phylogeny and carpology support the relation between two endemics – *B. bonus-henricus* from Alps and the West American *B*. *californicum*. The close relations between the Eurasian *B. virgatum* group and *B. capitatum*, supported by the carpology as well as the heterogeneity of a part of *Blitum* earlier considered within the genus *Monolepis*, require further investigation.

### General Conclusions Concerning the Divergence of Carpological Traits in the Subfam. Chenopodioideae

According to the latest molecular results the subfamily is divided into several clades: the tribes Dysphanieae, Chenopodieae (incl. Atripliceae), Anserineae, and Axyrideae [29]. Despite the high divergence of the subfamily members in life history and habit, there are many characteristics which support assigning them to a single group. The most appreciable traits are, as a rule, petiolate leaves with flat lamina (except *Chenopodium sancti-ambrosii* with terete leaves) and dense dichasial inflorescences [64], [101] often referred to as glomerules or clusters (the representatives of *Dysphanieae* often differ from other groups by the reduction of the cymes to solitary flowers). Small anthers (0.2–0.4 mm long) are typical for species of the whole subfamily. In fact the only carpological trait shared by all members of the subfamily is the abundant seed perisperm. In the case of other characteristics there are exceptions to any general rule, so it is necessary to consider the most common features within the Chenopodioideae.

#### 

##### Pericarp

Generally the parenchymatous pericarp is common to the entire *Chenopodioideae*. Usually it is undifferentiated. Only the genus *Axyris* (tribe Axyrideae) has a two-layered pericarp that splits into parenchymatous and sclereid layers. The supporting tissue is always visible in one of the two heterocarpous fruit types, whereas the other type usually lacks sclereids, which are a facultative characteristic in *Axyris mira* (for more see [102]). The presence of sclerenchyma in *Axyris* fruits is clearly an apomorphic feature of the entire subfamily Chenopodioideae. The one- or few-layered pericarp in the mature fruit is considered to be another ancestral trait that was transformed into a multilayered pericarp in many groups: Australian *Chenopodium* taxa earlier considered as genera *Rhagodia* and *Einadia*; *Chenopodium* sect. *Skottsbergia*, *Oxybasis macrosperma* and some genera of the Archiatriplex-clade. However, in a part of the Chenopodioideae (some Australian *Chenopodium*) the pericarp of one of the fruit types appears to be fleshy and attracts birds for dispersal. The dry fruits of *Chenopodium* can also be eaten by birds and are facultatively dispersed by endozoochory [103].

Both general trends in the evolution of the pericarp structure (development of a multilayered and differentiated fruit cover) are observed in taxa that are locally distributed or possess relatively small ranges while occurring on all continents.

##### Seed coat

It is clearly differentiated into a thick exotesta layer and thin endotegmen layer(s), with rare exceptions such as *Halimione* (Chenopodieae sensu novo), *Krascheninnikovia* and *Ceratocarpus* (Axyrideae) having two compressed layers of equal thickness. The testa is often crustaceous and dark; within gen. *Axyris* and the mostly annual *Atriplex*, however, there is an evident trend to develop a thin yellow testa. Both (dark and yellow) testa types of the seed coat are combined within an individual, resulting in the evident heterospermy and/or heterocarpy that are common especially in *Axyris* and many annual *Atriplex* [102], [104]–[107]. Usually the heterospermy involves differences in the shape, colour or weight of the seeds [108]. However, somatic seed polymorphisms in *Atriplex hortensis*, *A. sagittata* and *A. aucheri* can also be cryptic when both types of black seeds seem to be morphologically similar and have approximately the same weight [109], although with differing thickness of the testa [110]. The cryptic seed heterogeneity has now been found in several lineages within the subfamily. Trispermy – the highest degree of seed heteromorphism, expressed as three seed types combining the cryptic and evident kinds of seed heterogeneity – has evolved twice independently in some *Chenopodium* s. str., and *Atriplex*.

The locally evolved apomorphic traits in fruit/seed structure within Chenopodioideae seem to be as follows: (1) significant fusion of stylodia into a column (see also Skottsberg [111]) in two species of *Chenopodium* sect. *Skottsbergia*; (2) appearance of an equatorial keel on the seed (all clades except the Atriplex- and Archiatriplex-clades, and Axyrideae); (3) alveolation of the testa (*Chenopodium* s.str., *Chenopodiastrum*, *Oxybasis*, and *Blitum*; (4) drastic decrease in seed diameter and an almost straight embryo in Australian members of *Dysphania* as well as a pericarp with multilayered glandular or curved trichomes (in part of the tribe Dysphanieae); (4) unique hair-like outgrowths on the seed-coat cells (*Blitum atriplicinum*; *Blitum nuttallianum*), also found in the order Centrospermae in *Rivina* (Sukhorukov, unpubl.).

## Supporting Information

Appendix S1 (DOC)
