# Supplementary material for: Fruit and Seed Anatomy of Chenopodium and Related Genera (Chenopodioideae, Chenopodiaceae/Amaranthaceae): Implications for Evolution and Taxonomy
Source: PLoS One. 2013 Apr 23;8(4):e61906. doi: 10.1371/journal.pone.0061906 (PMC3633980; doi:10.1371/journal.pone.0061906)
Supplement: Appendix S1 — (DOC) [file pone.0061906.s001.doc]

Appendix S1. List of species investigated and their anatomical descriptions.

The species with unclear status that are not involved in the molecular analysis are listed below under the name *Chenopodium*. The species of the clades Atriplex, Archatriplex and Axyrideae have been described in previous articles (Sukhorukov 2005; 2011; Kadereit & al. 2010).

The fruit dimensions (diameter and thickness) correspond generally with the seed dimensions if the pericarp is relatively thin (up to 100 μm). Where the pericarp is thick the dimensions of the fruit and seed are recorded separately. Other uncommon characters are indicated where necessary.

*Blitum asiaticum* (Fisch. et C.A. Mey.) S. Fuentes, Uotila & Borsch: Russia, Yakutiya, delta of Lena river, Bulun, VII.1938, A.B. Nikolayev *90* (MW). — Fruit 1—1.2 mm in diameter, 0.4—0.5 mm thick. Pericarp ruptured easily, 1—layered, 20—40 μm thick. Seed red, keeled. Testa 7—10 μm thick, undulate, without stalactites; cotyledons obliquely oriented.

*B. atriplicinum* F. Muell.: Australia, NSW, McInsyre Brook, 22.IX.1944, C.T. White *12706* (LE). — Fruit 1.2—1.4 mm in diameter, ca. 0.9 mm thick. Pericarp tightly adhering to the seed and difficult to remove, 1—2(3)—layered. Seed dark red. Testa alveolate, 17—25 μm thick, with hair-like acicular outgrowths, without stalactites.

*B. bonus-henricus* (L.) Reichenb.: 1) Italy, Cuneo, VIII.1910, G. Gresino *s.n.* (LE); 2) [European Russia], Ryazan prov., Kasimov, 1964, N. Lazareva *372* (MW). — Fruit 1.6—2 mm in diameter, 1—1.1 mm thick. Pericarp scraped off, 1—3—layered, 30-50 μm thick, without papillae. Seed visually dark red (almost black), without keel. Testa 37—45 μm, smooth, without stalactites, cell protoplasts occupy half of the cell volume.

*B. californicum* S.Watson: 1) USA, California, San Francisco, V.1935, L.S. Rose *35155* (LE); 2) California, Goleta, IV.1958, H.M. Pollard *s.n.* (W-21418). — Fruit 1.6—1.9 mm in diameter, 1—1.3 mm thick. Pericarp difficult to remove from the seed, 1-3-layered, 50—120 μm thick, without papillae (only mamillate). Seed visually dark red (almost black), without keel. Testa 25—30 μm and 37—45 μm in different seed types, alveolate, without stalactites, protoplasts occupy half or more of the cell volume.

*B. capitatum* L.: 1) Canada, Mackenzie distr., Alexandra falls, VII.1951, W.H. Lewis *929* (W-17931); 2) [Canada], Manitoba, Blaeberry, VII.1955, A. & D. Löve *6768* (MHA). — Fruit ovoid, 0.9—1 mm long, 0.5—0.6 mm thick. Pericarp adhering to the seed coat, 1—2—layered, 5-12 μm thick. Seed visually dark red, with keel. Testa 12—15 μm, undulate, without stalactites.

*B. litvinovii* (Paulsen) S. Fuentes, Uotila & Borsch: 1) Afghanistan, Ghorat prov., VII.1962, K.H. Rechinger *19090* (W-6421); 2) Kyrghyzstan, Chüy Region, Panfilov distr., VII.2009, P. Uotila *47272* (MW). — Fruit ovoid, 0.8— 1 mm long, 0.5—0.6 mm thick. Pericarp adhering to the seed coat, 1—2—layered, 10—25 μm thick. Seed visually dark red, marginally with groove and two blunt keels. Testa 10—12 (first sampling) and 15—25 (second sampling) μm thick, alveolate, without stalactites.

*B. nuttallianum* Schult.: Canada, Alberta, Newcastle, VI.1971, W.G. D’Arcy *5591* (H). — Fruit 1.2 mm in diameter, 0.5—0.6 mm thick. Pericarp hardly scraped off the seed, 1—layered, 20—30 μm thick, without papillae. Seed red, without keel. Testa ca. 8 μm, with hair-like acicular outgrowths, without stalactites; cotyledons obliquely oriented.

*B. petiolare Link*: Algeria, 1968, V.P. Boczantsev *s.n.* (LE). — Fruit ovoid, ca. 1 mm long, 0.65—0.7 mm thick. Pericarp adhering to the seed coat, 1—2—layered, 5—15(20) μm thick. Seed visually dark red, with marginal groove and two blunt keels. Testa alveolate, 15—20 μm thick, without stalactites.

*B. spathulatum* (A.Gray) S. Fuentes, Uotila & Borsch: USA, California, Susanville, VIII.1983, I.Yu. Koropachinsky et al. *404* (MHA). — Fruit ovoid, 0.6—0.7 mm long, 0.4 mm thick. Pericarp ruptured easily, 1(2)—layered, 7—10 μm thick, papillae large, up to 100 μm. Seed red, ca. 0.5 mm long and 0.3 mm thick, without keel. Testa 10—12 μm thick, smooth, with stalactites; cotyledons obliquely oriented.

*Blitum virgatum* L.: 1) [Tadjikistan], Oy-Badym, VII.1933, Arsentyeva, Tekutyeva *66* (MW); 2) Mongolia, Khangay, Tsetserleg, VIII.1980, I.A. Gubanov *1051* (MW). — Fruit ovoid, 1—1.2 mm long, ca. 0.7 mm thick. Pericarp adhering to the seed coat, 1—2—layered, 5—10(15—20) μm thick. Seed visually dark red, with groove and two blunt keels on the margin. Testa undulate, 10—12 μm thick, without stalactites.

*Chenopodiastrum badachschanicum* (Tzvel.) S. Fuentes, Uotila & Borsch: 1) Turkestania [Kirghizia?], VII.1895, S. Korzhinsky *4638*, sub *C. hybridum* (LE); 2) NW Pakistan, Swat, VIII.1962, K.H. Rechinger *19544* (W). — Fruit 1.5—2 mm in diameter, 0.9—1.1 mm thick. Pericarp difficult to remove from the seed, 1—2—layered, 12—25 μm thick with small papillae easily losing turgidity. Seed visually black, without keel. Testa 110—130 μm thick, alveolate, with oblique stalactites.

*C. coronopus* (Moq.) S. Fuentes, Uotila & Borsch: [Spain, Canary], Tenerife, V.1855, ex herb. Hookerianum *s.n.* (K). — Fruit ca. 1 mm in diameter, 0.5 mm thick. Pericarp easily detached, 1—2—layered, with papillae up to 55 μm. Seed black, keeled. Testa 25—30 μm thick, undulate, with stalactites.

*C. hybridum* (L.) S. Fuentes, Uotila & Borsch: 1) [Russia], Voronezh prov., Kalach, IX.1946, N.N. Kaden *s.n.* (MW); 2) [Kazakhstan], Altay, Bukhtarma valley, Berel, IX.1932, A. Voronov *935* (MW); 3) [Russia], Tuva, Mugur-aksy, VIII.1973, Komkova *s.n.* (MW); 4) [Russia], Altay, Tenginskoye Lake, VIII.1984, I. Pshenichnaya, G. Liventsova *s.n.* (MW); 5) Moscow, Presnya, VII-X.2008, A. Sukhorukov *s.n*. (MW). — Fruit 1.4—2 mm in diameter, ca. 1 mm thick. Pericarp difficult to remove from the seed, 1—2—layered, 12—20 μm thick with small papillae easily losing turgidity. Seed visually black, without keel. Testa 70—110 μm and 30—55 μm thick in different seed types, alveolate, with oblique stalactites.

*C. murale* (L.) S. Fuentes, Uotila & Borsch: Israel, Tel-Aviv–Yaffo, XII.2008, A. Sukhorukov *s.n.* (MW). — Fruit 1.3—1.4 mm in diameter, 0.6—0.7 mm thick. Pericarp difficult to remove from the seed, 1(2)—layered, 10—20 μm thick, papillae up to 75 μm, all cells with dark protoplasts and starch grains. Seed visually black, sharply keeled. Testa 32—40 μm thick, undulate, with stalactites.

*C. simplex* (Torrey) S. Fuentes, Uotila & Borsch: 1) Canada, Manitoba, Morden, VIII.1951, H.J. Scoggan *10581* sub *C. hybridum* var. *gigantospermum* (LE); 2) USA, Michigan, X.1997, J. Walter *s.n.* (W). — Fruit 1.7—1.8 mm in diameter, ca. 1 mm thick. Pericarp easily detached and ruptured, 1—2—layered, 25—50 μm thick, with cratered surface. Seed visually black, slightly keeled. Testa ca. 75 μm thick, with oblique stalactites.

*Chenopodium acerifolium* Andrz.: 1) Russia, Moscow, Bitsa, IX.1998, A. Sukhorukov *s.n.* (MW); 2) Russia, Volgograd prov., Krasnaya Sloboda, IX.2005, A. Sukhorukov *s.n.* (MW). — Fruit 1—1.3 mm in diameter, 0.5—0.7 mm thick. Pericarp scraped off the seed, 1(2) —layered, pericarp cells (if not papillous) 10—25 μm thick, papillae up to 35 μm. Seed visually black, without keel. Testa 20—25 μm, smooth, with stalactites.

*C. acuminatum* Willd.: 1) Kazakhstan, Taldy-Kurgan distr., VI.1928, V.I. Smirnov *57* (MW); 2) Mongolia, Ubsunur distr., Baga-Nur, VIII.1979, I.A. Gubanov *8129* (MW). — Fruit 1 mm in diameter, 0.4—0.5 mm thick. Pericarp scraped off the seed, 1—(2)—layered, 5—12(20) μm, with papillae up to 35 μm, its cells often with abundant starch grains. Seed visually black, keeled. Testa 17—22 μm, smooth or slightly undulate, with stalactites.

*C. album* L. s.str.: [European Russia], Tambov prov., Umyot distr., Yadrovka, VIII.2009, A. Sukhorukov *s.n*. (MW). — Fruit 1.3-1.5 mm in diameter, 0.6—0.7 mm thick. Pericarp scraped off the seed, 1—2(3)—layered, with papillae up to 55 μm. Seed visually black, slightly keeled (yellow seeds were not observed in the present study). Testa 50 μm and 17—25 μm in different seed types, smooth, with stalactites.

*C. anidiophyllum* Aellen: Australia, New South Wales, IV.1967, J. Blackbern (LE). — Fruit 1.5 mm in diameter, 0.6 mm thick. Pericarp scraped off the seed, 1—2(3)—layered, 10—20 μm, with papillae up to 55 μm. Seed visually black, without keel. Testa 35—40 μm, smooth, with stalactites.

*C. antarcticum* Benth. & Hook.f.: Argentina, Tierra del Fuego, Bahia San Sebastian, I.1968, D.M. Moore *1481* (H-1538267). — Fruit ovoid, 0.8—0.9 mm in diameter, 0.5—0.6 mm thick. Pericarp (or its outer layer) easily detached and ruptured, 2—3—layered, 25—60 μm thick, without papillae (mamillate). The subepidermal layer(s) often brownish. Seed visually dark red (almost black), without keel. Testa slightly undulate, 15—20 μm thick, without stalactites. Carpologically very close to *Oxybasis*.

*C. atripliciforme* Murr: Afghanistan, Konar, Dewagal Darrah, VIII.1973, O. Anders *11078* (W-06301). — Fruit 1.3—1.4 mm in diameter, 0.6—0.7 mm thick. Pericarp scraped off the seed, 1-2-layered, 7—15 μm thick, with dark protoplast, papillae up to 50 μm. Seed visually black, without keel. Testa 35—40 μm, smooth or slightly undulate, with stalactites.

*C. atrovirens* Rydb.: USA, Nevada, Washoe co., VII.2001, A. Thiem *13684* (W-08389). — Fruit 1—1.1 mm in diameter, ca. 0.5 mm thick. Pericarp easily detached from the seed and ruptured, 1(2)—layered, with small papillae 8—15 μm, dark coloured and with abundant starch grains. Seed visually black, without keel. Testa 35—40 μm, smooth, with stalactites.

*C. berlandieri* Moq.: USA, Colorado, IX.1900, F.K. Vreeland *661* (K). — Fruit 1.2—1.5 mm in diameter, 0.7—0.8 mm thick. Pericarp scraped off the seed, 1—layered, 25—50 μm thick, with spongy (mamillate) cells. Seed visually black, without keel. Testa 50—65 μm, undulate, with stalactites.

*C. boscianum* Moq.: USA, Illinois, St. Clair Co., X.1918, M. Greeman *4033* (LE). — Fruit 1.2—1.4 mm in diameter, ca. 0.9—1 mm thick. Pericarp easily detached from the seed and ruptured, 1(2)—layered, dark coloured, with papillae up to 55 μm. Seed visually black, without keel. Testa 65—70 μm, smooth, with stalactites.

*C. carnosulum* Moq.: [Argentine], Tierra del Fuego, Viamonte, III.1936, Y. Mexia *7960* (BM). — Fruit ca. 1 mm in diameter, 0.6—0.7 mm thick. Pericarp easily detached and ruptured, 1—2—layered, up to 30 μm thick, with papillae up to 50 μm, with abundant starch grains. Seed visually black, without keel. Testa 30—35 μm, smooth, with stalactites.

*C. crusoeanum* Skottsb.: [Chile], Juan Fernandez, Masatierra, I.1917, C. & I. Skottsberg *227* (E). — Fruit 1.3—1.4 m in diameter. Pericarp multilayered, rough, with prominent longitudinal furrows and ribs, up to 550 μm thick, with maximum thickness near the column base, without papillae, tightly adhering to the seed and difficult to remove. The (sub)epidermal layers often contain starch grains. Seed 1.2—1.3 mm in diameter, ca. 0.6 mm thick, visually black. Testa 37—50 μm, with stalactites.

*C. desiccatum* A. Nelson: N Mexico, 1851–1852, C. Wright 1733 (K). — Fruit 1.1—1.3 mm in diameter, ca. 0.7 mm thick. Pericarp easily detached from the seed and ruptured, 1(2)—layered, 7—15 μm thick, with small papillae; protoplasts of the cells with starch grains. Seed visually black, slightly keeled. Testa 50—55 μm, smooth, with stalactites.

*C. detestans* Kirk: New Zealand, South Island, D. Petrie *157* (LE). — Fruit 1—1.2 mm in diameter, ca. 0.6 mm thick. Pericarp scraped off the seed, 1—2(3)—layered, with papillae up to 30 μm. Seed visually black, without keel. Testa 40—45 μm, smooth, with stalactites.

*C. fasciculosum* Aellen: Kenya, North Kavirondo distr., Mt. Elgon, III.1977, S.S. Hooper, C.C. Townsend *1379* (LE). — Fruit 1.5 mm in diameter, 0.65 mm thick. Pericarp difficult to remove from the seed, 1—2—layered, 7—15 μm thick. Seed visually black, keeled. Testa 37—40 μm (in central part increasing to 75 μm), smooth or undulate, with oblique stalactites.

*C. ficifolium* Smith: Moscow, Pechatniki, X.1996, A. Sukhorukov *s.n.* (MW). — Fruit 1.2 mm in diameter, 0.65—0.8 mm thick. Pericarp (or its outer layer) easily detached, 1—2—layered, 10—20 μm thick, with papillae up to 40 μm. Seed visually black, slightly keeled. Testa undulate, ca. 15 μm, with stalactites.

*C. fremontii* S. Watson: [USA], Arizona, Greenlee co., X.1960, C.T. Mason, R.A. Kidwell *1888* (W-20757). — Fruit 1.1—1.3 mm in diameter, 0.65—0.8 mm thick. Pericarp easily detached from the seed (or subepidermal layer), 1—2—layered, dark coloured, all cells papillate, up to 30 μm. Seed visually black, without keel. Testa smooth or slightly undulate, 60—75 μm, with stalactites.

*C. frutescens* C.A. Mey.: Mongolia, Ubsu-Nur aymak, Naran-bulak, IX.1984, I.A. Gubanov *1397* (MW). — Fruit ca. 1.5 mm in diameter, 0.9 mm thick. Pericarp scraped off the seed, 1—2(3)-layered, 25—50 μm thick, cells of outer (or single) layer spongy, but not papillate. Seed visually black, without keel. Testa smooth or slightly undulate, 25—30 μm thick, with stalactites.

*C. giganteum* D. Don: [Plantae cultae], Moscow, III.2011; origin of material: Nepal, Trekking route Ghorepani-Nayapul, IX.2009, A. Sukhorukov *s.n.* (MW). — Fruit 1.2—1.4 mm in diameter, 0.65—0.8 mm thick. Pericarp (or its outer layer) easily scraped off, 1-2-layered, 20—30 μm thick, papillae up to 75 μm. Seed visually black, slightly keeled. Testa smooth or undulate, ca. 25 μm thick, with stalactites.

*C. gracilispicum* H.W. Kung: [Japan], Sanbagawa, Gunma pref., IX.1979, J. Murata *1891* (MHA). — Fruit 1—1.1 mm in diameter, 0.55—0.65 mm thick. Pericarp easily detached and ruptured, 1—2—layered, 12—20 μm thick, with papillae up to 40 μm. Seed visually black, keeled. Testa 37—50 μm, alveolate, with oblique stalactites.

*C. gubanovii* Sukhor.: [Russia], Tuva Republic, Ovyursky distr., VIII.1976, E. Korotkova, V. Nikolayeva *491* (MW). — Fruit 0.6—0.7 mm in diameter, ca. 0.35 mm thick. Pericarp scraped off, 1—layered, 3—10 μm thick, without papillae. Seed red, keeled. Testa smooth or pitted, 12—15 μm thick, with stalactites. Carpologically C. *gubanovii* is very close to *Oxybasis*.

*C. hederiforme* Aellen: South Rodesia, Limpopo, V.1959, R.B. Drummond *s.n.* (K). – Fruit 1—1.3 mm in diameter, 0.6—0.7 mm thick. Pericarp scraped off the seed coat, 1—2—layered, thin (to 15 μm), but with small papillae to 35 μm. Its cells contain abundant starch grain. Seed visually reddish, keeled. Testa 20—25 μm, smooth, with stalactites.

*C. hians* Standl.: USA, Oregon, Columbia river, [anno] 1860, Dr. Lyall *s.n.* (K). — Fruit 1.4—1.5 mm in diameter, 0.8—0.9 mm thick. Pericarp scraped off the seed coat, 1—2—layered, thin but with papillae up to 60—70 μm high. Seed visually black, not keeled. Testa 45—50 μm, smooth, with stalactites.

*C. hircinum* Schrad.: [Switzerland], Basel, IX.1939, P. Aellen *s.n.* (MW). — Fruits 1.5 mm in diameter, 0.8—0.9 mm thick. Pericarp easily scraped off seed, 1(2)—layered, 30-65 μm thick, cells with convex outer walls (mamillate) but without papillae. Seed visually black, without keel. Testa 32—37 μm thick, smooth or slightly undulate, with stalactites.

*C. iljinii* Golosk.: [Kazakhstan], Talas-alatau, VIII.1931, N.V. Pavlov *1015* (MW). — Fruit 1—1.2 mm in diameter, 0.5—0.6 mm thick. Pericarp scraped off the seed, 1(2)—layered, 10—25 μm thick, rough but without pronounced papillae. Seed visually black, without keel. Testa smooth, ca. 30 μm thick, with stalactites.

*C. incanum* (S. Watson) A. Heller: USA, Arizona, Camp Lowell, VI.1882, C.G. Pringle *s.n.* (K). — Fruit ca. 1.2 mm in diameter, 0.7 mm thick. Pericarp scraped off the seed coat, 1—2—layered, papillate. Seed visually black, slightly keeled. Testa 45—55 μm, smooth, with stalactites.

*C. karoi* Aellen: 1) [Russia, Tyva], between Bey-hem and Ulu-hem, VIII.1916, G. Miklashevskaya *s.n.* (LE); 2) Afghanistan, Sang Lech, VII.1937, W. Koelz *12647* (W-06204). — Fruit 1.1—1.3 mm in diameter, 0.5—0.6 mm thick. Pericarp scraped off, 1(2)—layered, rough, 25—50 μm thick, with small papillae. The cells of the pericarp of the first sample contain abundant starch grains. Seed black, without keel. Testa 20—25 μm thick, smooth or undulate, with stalactites.

*C. mexicanum* Moq.: Mexico, V.1904, C.G. Pringle 8984 (BM). — (Fruits with horizontal seed embryo were examined): Fruit 1.1-1.2 mm in diameter, 0.55—0.65 mm thick. Pericarp (or its outer layer) easily detached and ruptured, 2—3—layered, 35—60 μm, without papillae. Seed visually red, without keel. Testa smooth or undulate, 20—25 μm, with stalactites.

*C. mucronatum* Thunb.: Lesotho, Maseru distr., II.1987, J. Backeus 2128 (E). — Fruit ca. 1.4 mm in diameter, 0.8 mm thick. Pericarp scraped off the seed, 1(2)—layered, minute (5—7 μm) with papillae up to 30 μm. Seed black, without keel. Testa 35—40 μm thick, slightly undulate, with stalactites.

*C. nesodendron* Skottsb.: Chile, Juan Fernandez Archipelago, Isla Mas Afuera, II.1917, C. & I. Skottsberg *523* (K-000583184). — Fruit 1.6 mm in diameter, ca. 1 mm thick. Pericarp scraped off the seed, with prominent longitudinal furrows and ribs, multilayered and robust (300—600 μm) particularly at the apex of the fruit, without papillae. Seed visually black, 1.2—1.3 mm in diameter, ca. 0.8 mm thick, without keel. Testa undulate, 45—55 μm thick, with stalactites.

*C. nevadense* Standl.: USA, Nevada, Austin, IX.1937, W.H. Henning *80* (LE). — Fruit 0.9—1 mm in diameter, 0.5—0.6 mm thick. Pericarp easily detached from the seed and ruptured, 1(2)—layered, 10—20 μm thick, with small papillae; protoplasts of all cells with starch grains. Seed visually black, without keel. Testa 12—15 μm, smooth, with stalactites.

*C. novopokrovskyanum* (Aellen) Uotila: Afghanistan, Farqar, VI.1996, P. Furse *8192* (W-10316). — Fruit 1—1.2 mm in diameter, 0.5—0.6 mm thick. Pericarp scraped off the seed, 1—2—layered, 7-15 μm thick, with papillae up to 30 μm. Seed visually black, without keel. Testa 35—40 μm thick, smooth or slightly undulate, with stalactites.

*Chenopodium nutans* (R.Br.) S. Fuentes & Borsch: Australia, Canberra, V.2002, S.A. Afonin *s.n.* (MW). — Pigmented fruit ca. 4 mm long, seed located in its basal part; pericarp papillous, fleshy, orange, consisting of 6-8 layers; seed 1.5 mm in diameter, testa 50—60 μm thick, undulate, with stalactites. Unpigmented fruits much smaller in size (ca. 1.5—2 mm in diameter), with thin 1—2—layered or 2—4—layered slightly spongy papillate pericarp; seed ca 1 mm in diameter, 0.5-0.6 mm thick, 40—50 μm thick, undulate or alveolate, with stalactites.

*C. oahuense* (Meyen) Aellen: 1) USA, Hawaii, VIII.1927, O. Degener 17902 (W-15572); 2) USA, Hawaii, Anapuka, IV.1928, O. Degener *18175* (LE) both as *C. sandwicheum* Moq. — Fruit 1.2—1.5 mm in diameter, 0.6—0.7 mm thick. Pericarp scraped off the seed, 1—3—layered, 25—40 μm thick, with papillae up to 120 μm. Seed visually black, without keel. Testa 30—40 μm thick, smooth or undulate, with stalactites.

*C. opulifolium* Schrad.: Israel, Jerusalem, Giv’at Ram, X.2012, A. Sukhorukov, M. Kushunina *215* (MW). — Fruit 1.2—1.4 mm in diameter, 0.6—0.7 mm thick. Pericarp scraped off the seed, 1—2(3)—layered, with papillae up to 40 μm. Seed visually black, without keel. Testa 35—40 μm thick, smooth, with stalactites.

*C. pallescens* Standl.: USA, New Mexico, Roswell, VIII.1900. F.S. & E.S. Earle *326* (K). — Fruit 1.5—1.7 mm in diameter, 0.9—1.1 mm thick. Pericarp adhering to the seed coat, 2—3(4)—layered, 50—70 μm thick, papillate; all cells dark coloured. Seed visually black, slightly keeled. Testa 65—75 μm, smooth, with stalactites.

*Chenopodium pallidicaule* Aellen: Bolivia, IV.1898, G. Mandon *1021* (K). — Fruit 1.3—1.4 mm in diameter, ca. 1 mm thick. Pericarp scraped off the seed, 1(2)—layered, with small papillae or almost smooth, very thin (5—15) μm thick. Seed visually brownish, without keel or slightly acute. Testa 15—20 μm thick, smooth, with stalactites.

*C. pallidum* Moq.: Central Nepal, Mustang prov., Jomosom-Mukhtinat, IX.2009, A. Sukhorukov *s.n.* (MW). — Fruit 1.3—1.4 mm in diameter, ca. 0.6 mm thick. Pericarp scraped off the seed, 1—2—layered, 7—10 μm thick, papillae up to 25 μm. Seed visually black, without keel or slightly acute. Testa 32—37 μm thick, smooth, with stalactites.

*C. pamiricum* Iljin: 1) Mongolia, Khangay, VIII.1924, N.V. Pavlov *522* (MW); 2) Mongolia, inter oppidum Arvajcher et pagum Gučin-us, VIII.1966, J. Soják, V. Vašak *6289* (Pr). — Fruits (and seeds) of two types: The first ca. 1 mm in diameter, 0.5 mm thick; pericarp adhering to the seed, 1—layered, 5—15 μm thick, with papillae up to 20(25) μm thick; seed blackish, with keel, testa 17—25 μm thick, with stalactites. Fruit of the second type ovoid, 1—1.2 mm long, ca. 0.5 mm thick; pericarp adhering to the seed, 1—layered, without papillae; seed yellow, without keel, testa 5—8 μm, without stalactites.

*C. paniculatum* Hook.: Bolivia, Catania, XI.1911, O. Buchtien *s.n.* (BM). — Fruit 1.4—1.5 mm in diameter, ca. 1 mm thick. Pericarp scraped off the seed, 1—layered, papillae up to 100 μm. Seed visually black, without keel. Testa 35—55 μm thick, smooth, with stalactites.

*Chenopodium parabolicum* (R.Br.) S. Fuentes & Borsch: South Australia, Murray, VII.1985, D.J.E. Whibley *9879* (H-1689518). – (Only the red fruits were examined): Fruits ca. 4 mm. Pericarp coloured, more than 150 μm thick, 4—12-layered, without papillae, scraped off the seed. Seed 1.6—1.7 mm in diameter, visually black, without keel. Testa 50—65 μm, slightly undulate, with stalactites.

*C. phillipsianum* Aellen: Plantae cultae, Austria, Vienna, Bot. Garden, IV.2003, J. Walter *s.n.* (W-13897). Origin: South Africa. — Fruit 1.5 mm in diameter, 0.6—0.7 mm thick. Pericarp scraped off the seed, 1(2)—layered, 10-15 μm thick, papillae up to 30 μm. Seed visually black, without keel. Testa 50-65 μm thick, undulate, with stalactites.

*C. pratericola* Rydb.: 1) USA, Nevada, Wassuk range, VIII.1938, W.A. Archer *6841* (LE); 2) USA, South Dakota, VIII.1950, F. Petrich *s.n.* (W-14240). — Fruit 1—1.3 mm in diameter, 0.5-0.6 mm thick. Pericarp ruptured easily, 1—2—layered, 10—20 μm thick, with papillae up to 30 μm thick; the pericarp cells with starch grains. Seed black, marginally thickened and slightly keeled. Testa ca. 50 and 70 μm thick (respectively in the two fruit types), smooth, with stalactites.

*C. preissii* (Moq.) Diels: South Australia, Eyre Peninsula, XI.1964, C.R. Alcock *s.n*. (H-1688951). — Reddish fruits ca. 2.5 mm in diameter. Pericarp scraped off the seed, multilayered, (25—40)100—400 μm thick, without papillae. Seed visually black, 1.4—1.6 mm in diameter, 0.8—0.9 mm thick, without keel. Testa 35—40 μm, slightly undulate, with stalactites. Unpigmented fruits smaller, 1.7—1.8 mm in diameter. Pericarp 3—5—layered, (40)100—200 μm thick. Seed black, 1.6 mm in diameter,

*C. quinoa* Willd.: [Plantae cultae], Finland, Helsinki, Kaisaniemi, Botanical Garden, X.1983, P. Uotila *33323* (H-1188137). — Fruit 2—2.5 mm in diameter, 1—1.1 mm thick. Pericarp scraped off the seed, 20—40 μm thick, papillae up to 100 μm thick, 1—2—layered. Seed yellow (black seeds were not seen), without keel. Testa 12—15 μm thick, smooth or slightly undulate.

*C. sanctae-clarae* Johow: Chile, Juan Fernandez Archipelago, Isla Masatierra, XII.2003, M.F. Gardner & al. *85* (E). — Fruit 1.8—2 mm in diameter, ca. 1.3 mm thick. Pericarp multilayered, rough, with prominent longitudinal furrows and ribs, 150—350 μm thick, with maximum thickness near the column base (up to 600 μm), without papillae, tightly adhering to the seed and difficult to remove. The (sub)epidermal layers often contain the starch grains. Seed ca. 1.5 mm in diameter, 0.65—0.8 mm thick, black, rough in its upper half and concave apically. Testa ca. 50 μm, with stalactites.

*C. sancti-ambrosii* Scottsb.: Chile, San Ambrosio Island, XI.1960, G. Kuschel (K). — Fruit 1—1.2 mm in diameter, ca. 0.6 mm thick. Pericarp scraped off the seed, 20—40 μm thick, 2—3—layered with compressed inner layer(s), mamillate. Seed black, without keel. Testa 40–45 μm thick, undulate, with stalactites.

*C. sosnovskyi* Kapeller: [Transcaucasus], Karabagh, IX.1934, V.A. & I.P. Petrov *s.n.* (MW). — Fruit 1.2—1.5 mm in diameter, ca. 0.6 mm thick. Pericarp scraped off the seed, 1—2—layered, 50 μm thick, with papillae up to 25 μm. Seed visually black, without keel. Testa ca. 50 μm thick, undulate, with stalactites.

*C. standleyanum* Aellen: USA, Missouri, Vale, VIII.1927, B.F. Bush 11456 (K). — Fruit 1.3—1.5 mm in diameter, 0.7—0.8 mm thick. Pericarp easily detached from the seed and ruptured, 1(2)—layered, dark coloured, up to 40 μm, with tiny papillae. Seed visually black, slightly keeled. Testa 50—55 μm, smooth, with stalactites.

*C. strictum*: Moscow, Losiny Ostrov, IX.1997, Yu.A. Nasimovich *s.n.* (MW). — Fruit 1.2—1.4 mm in diameter, ca. 0.7 mm thick. Pericarp scraped off the seed, 1—2—layered, 10—20 μm thick, with papillae up to 30 μm; the protoplast of all cells with abundant starch grains. Seed visually black, without keel. Testa 25—30 μm thick, smooth or slightly undulate, with stalactites.

*C. subglabrum* A. Nelson: USA, Upper Missouri, C.A. Geyan *31* (K). — Fruit 1.2—1.5 mm in diameter, 0.7—0.8 mm thick. Pericarp easily ruptured, 2—3—layered, to 60 μm thick, with small papillae. Seed visually black, without keel. Testa 85—100 μm thick, smooth or slightly undulate, with stalactites.

*C. ugandae* (Aellen) Aellen: [Tanzania], Kilimanjaro, XI.1893, G. Volkens (E). — Fruit 1.1—1.3 mm in diameter, ca. 0.7 mm thick. Pericarp scraped off the seed, 1—2—layered, cells of outer (or single) layer spongy, (25)35—55 μm thick, the protoplast of cells brown. Seed visually black, without keel. Testa 35—40 μm thick, smooth, with stalactites.

*C. vulvaria* L.: 1) Kazakhstan, Talas Alatau, VIII.1931, N.V. Pavlov *s.n.* (MW); 2) Turkmenistan, Kopet-Dagh, Vannovskoye, VII.1942, V. Alyokhin *s.n.* (MW). — Fruit 1—1.2 mm in diameter, ca. 0.6 mm thick. Pericarp scraped off the seed, 1—2—layered, 5—150 μm thick with papillae up to 30 μm thick; all cells with dark-coloured protoplasts. Seed black, without keel. Testa 25 μm thick, smooth, with stalactites.

*C. wolffii* Simonk. (*Oxybasis glauca* group): Transsilvania, Torda [Romania, Turda], without date, G. & J. Wolff *1018* (MW). — (Only fruits containing seeds with horizontal embryo were examined). Fruit 0.7—0.9 mm in diameter, 0.5—0.55 mm thick. Pericarp (or its outer layer) easily detached and ruptured, 1—3—layered, 20—30 μm, without papillae. Seed visually red, without keel. Testa smooth or undulate, 17—22 μm thick, with stalactites.

*Cycloloma atriplicifolium* (Spreng.) J.M. Coult.: 1) USA, California, Huntington Beach, VII.1932, L.M. Booth *1343* (MHA); 2) Canada, Manitoba, Souris distr., IX.1960, B. Boiwin *13974* (MW). — Fruit 1.5—1.6 mm in diameter, 0.7—0.8 mm thick. Pericarp adhering to the perianth at its basal part, but scraped off from perianth as well as from the seed, 1—2—layered, up to 25 μm thick, with long curved and short (100-150 μm) glandular hairs. Seed black, without keel, concave apically. Testa 32—45 μm thick, alveolate, without stalactites.

*Dysphania ambrosioides* (L.) Mosyakin & Clemants: Israel, Jerusalem area, XII.2007, A. Danin, A. Sukhorukov *s.n.* (MW). — Fruit 0.7 mm in diameter, 0.5—0.6 mm thick. Pericarp scraped off the seed, 1(2)—layered, 5—15 μm thick, glandular hairs 100-130 μm. Seed dark red or blackish, without keel. Testa 12—15 μm thick, smooth, without stalactites.

*D. anthelmintica* (L.) Mosyakin & Clemants: USA, Jersey city, X.1908, H. Dautin *s.n.* (W-24531). — Fruit 0.7—0.8 mm in diameter, ca. 0.5-0.55 mm thick. Pericarp scraped off the seed, 1(2)—layered, 5—15 μm thick, glandular hairs 100—130 μm. Seed dark red or blackish, without keel. Testa 12—15 μm thick, smooth, without stalactites.

*D. bonariensis* (Hook.f.) Mosyakin & Clemants ex Sukhor.: South America [without precise location; anonym] (W). — Fruit ovoid, 1.3—1.5 mm long, 0.85—0.9 mm thick. Pericarp scraped off the seed, very thin (6-10 μm), 1—layered, glandular hairs 100—130 μm. Seed reddish, without keel. Testa 20—25 μm thick, undulate, without stalactites.

*D.botrys* (L.) Mosyakin & Clemants: 1) [China, Xinjang] Kuldsha, 1875, Larionov (MW); 2) Moldavia, Dubossary, VIII.1947, V.N. Andreev *91* (MW). — Fruit 0.6—0.8 mm in diameter, 0.5—0.6 mm thick. Pericarp scraped off the seed, 1(2)—layered, 5—12 μm thick, with tiny papillae up to 20 μm. Seed blackish, with small keel. Testa 10—12 μm thick, smooth, without stalactites.

*D. carinata* (R.Br.) Mosyakin & Clemants: 1) Tschechien, Mähren, IX.1896, Makovsky *s.n.* (W-17958); 2) Bohemia centr., Praha, VIII.1926, J. Rohlena *204* (MW). — Fruit 0.5—0.6 mm, 0.3—0.4 mm thick. Pericarp scraped off the seed, 1(2)—layered, 5—7 μm thick, with tiny papillae up to 15 μm. Seed reddish, without keel. Testa 8—12 μm thick, smooth, without stalactites.

*D. chilensis* (Schrad.) Mosyakin & Clemants: 1) [Bolivia], La Paz, 1889, N.L. Britton, H.H. Rusby *52* (LE); 2) Argentine, Mendza, XI.1926, D.O. King *s.n.* (BM). — Fruit 1—1.1 mm in diameter, 0.7—0.8 mm thick. Pericarp scraped off the seed, 1—2—layered, 7—17 μm thick, glandular hairs 100-130 μm. Seed reddish, without keel. Testa ca. 20 μm thick, smooth, without stalactites.

*D. congolana* (Hauman) Mosyakin & Clemants: Cameroon, Bamenda distr., 1958, F.N. Hopper *2079* (K). — Fruit ca. 1 mm in diameter, 0.6—0.7 mm thick. Pericarp scraped off the seed, 1—2—layered, 15—25 μm thick, without papillae. Seed reddish, without keel. Testa ca. 15 μm thick, smooth, without stalactites.

*D. cristata* (F. Muell.) Mosyakin & Clemants: Australia, Eyre Peninsula, VIII.1967, H. Eichler *19188* (W-2905). — Fruit 0.6—0.65 mm in diameter, 0.45—0.5 mm thick. Pericarp scraped off the seed, 1(2)—layered, 3-7 μm thick, with papillae up to 25—30 μm. Seed reddish, keeled. Testa 8—12 μm thick, undulate, without stalactites.

*D. graveolens* (Willd.) Mosyakin & Clemants: 1) USA, Arizona, anonym *s.n.* (LE); 2) Mexico, Oaxaca, X.1991, C. Romero & al. *8* (BM). — Fruit 0.7—0.8 mm in diameter, 0.6—0.65 mm thick. Pericarp scraped off the seed, 1(2)—layered, 3—7 μm thick, with papillae up to 25 μm. Seed blackish, keeled. Testa 10—15 μm, smooth, without stalactites.

*D. littoralis* R.Br.: Australia, New South Wales, Buckeroo Mtn., IX.1988, W. Greuter *s.n.* (B). — Fruit ovoid, 0.35—0.4 mm long, 0.22—0.27 mm thick. Pericarp scraped off the seed, 1—layered, 5—8 μm thick, without papillae. Seed blackish, with small groove. Testa ca. 7 μm thick, without stalactites.

*D. multifida* (L.) Mosyakin & Clemants: 1) USA, California, San Francisco co., XI.1932, L.S. Rose *32678* (MW); 2) [Italy], Castello del Valentino, 17.XI.1954, M. Calduch *s.n.* (BC-128952). — Fruit ovoid, 0.9—1.2 mm long, 0.65—0.7 mm thick. Pericarp scraped off the seed, 1—layered, 5—10 μm thick, bearing multicellular glandular hairs (100—150 μm) with large terminal cell. Seed reddish, without keel. Testa 15—20 μm, smooth or slightly undulate, without stalactites.

*D. nepalensis* (Colla) Mosyakin & Clemants: 1) Nepal, Marpha vill., IX.2009, A. Sukhorukov (MW); 2) West Nepal, Jumla vill., X.2010, A. Sukhorukov *s.n.* (MW). — Fruit 0.7—0.8 mm in diameter, 0.45—0.5 mm thick. Pericarp scraped off the seed, 1—layered, 3—7 μm thick, with papillae up to 25 μm. Seed blackish, with small keel. Testa 15—20 μm thick, without stalactites.

*D. procera* (Hochst. ex Moq.) Mosyakin & Clemants: [SE Africa], Nyasaland. 1891, J. Buchanan *845* (LE). — Fruit 0.9—1 mm in diameter, 0.6—0.7 mm thick. Pericarp scraped off the seed, 5—15 μm thick, 1(2)—layered, papillae up to 20 μm. Seed dark red, without keel. Testa 18—20 μm thick, smooth, without stalactites.

*D. pseudomultiflora* (Murr) Verloove & Lambinon: South Africa, [Eastern] Cape, Uitenhage, [without date], leg. Zeyher *103/4* (W-18377). — Fruit ca. 0.8 mm long, 0.6 mm thick. Pericarp scraped off the seed, 5—15 μm thick, 1(2)—layered, with tiny papillae. Seed reddish, without keel. Testa ca. 10 μm thick, smooth, without stalactites.

*D. pumilio* (R. Br.) Mosyakin & Clemants: Spain, Toledo, IX.1982, A. Zubizarreta *24034* (MHA). — Fruit 0.5—0.55 mm in diameter, ca 0.35 mm thick. Pericarp scraped off the seed, 5—7 μm thick, 1—2—layered, without papillae. Seed reddish, keeled. Testa 7—10 μm thick, smooth, without stalactites.

*D. schraderiana* (Schult.) Mosyakin & Clemants: Moscow, Vorobyovy Gory, Botanical Garden, IX.2008, Yu.E. Alexeev *s.n.* (MW). — Fruit ca. 0.8 mm in diameter, 0.55—0.65 mm thick. Pericarp scraped off the seed, 5—15 μm thick, 1(2)—layered, papillae up to 25 μm. Seed reddish, without keel. Testa ca. 15 μm, undulate, without stalactites.

*D. tomentosa* (Thouars) Mosyakin & Clemants: Tristan da Cunha, II.1939, L. Cuken (BM). — Fruit ca. 1 mm in diameter, 0.5-0.6 mm thick. Pericarp easily ruptured, 1(2)—layered, up to 15 μm thick, glandular hairs 100—130 μm. Seed reddish, without keel. Testa 13—17 μm thick, smooth, without stalactites.

*Lipandra polysperma* (L.) S. Fuentes, Uotila & Borsch: 1) [Russia], Kemerovo prov., Izhmorsky distr., Novyi Svet vill., VIII.1990, I.M. Krasnoborov *s.n.* (MW); 2) [European Russia], Tambov prov., Umyot distr., Yadrovka, IX.2002, A. Sukhorukov *s.n.* (MW). — Fruit 1—1.2 mm in diameter, ca. 0.6 mm thick. Pericarp scraped off the seed easily, 1—2—layered, 35—45 μm thick, without papillae (only mamillate). Seed visually black, without keel. Testa 25—35 μm thick, smooth or undulate, with stalactites.

*Micromonolepis pusilla* (Torr.) Ulbr.: 1) USA, Oregon, Malheur co., VI.1959, A. Cronquist *8420* (LE); 2) USA, Nevada, Nye co., V.2004, A. Tiehm *14517* (W-10513). — Fruit 0.6—0.7 mm in diameter, 0.35-0.45 mm thick. Pericarp ruptured easily, 1—2-layered, 5—15 μm thick (papillae up to 50 μm). Seed red, without keel. Testa 10—15 μm thick, smooth or slightly undulate, with stalactites; cotyledons obliquely oriented.

*Oxybasis chenopodioides* (L.) S. Fuentes, Uotila & Borsch: 1) Kenya, X.1976, T. Haye *153* (K); 2) Kazakhstan, Kzyl-Orda prov., 25 km NW Novokazalinsk, X.2004, A. Sukhorukov *34* (MW). — Fruit 0.6—0.8 mm in diameter, 0.35—0.45 mm thick. Pericarp (or its outer layer) easily detached and ruptured, 1—2—layered, up to 30 μm thick, without papillae. Seed visually red, without keel. Testa 10—15 μm thick, smooth, with stalactites.

*O. glauca* (L.) S. Fuentes, Uotila & Borsch: 1) Kyrghyzstan, Tersky Alatau, Pokrovka, VIII.1948, L. Sobolev *s.n.* (MW); 2) Moscow, Pechatniki, IX-X. 2008, A. Sukhorukov *s.n.* (MW). — (Fruits with both vertical and horizontal seed embryo were examined): Fruit 0.7—0.9 mm in diameter, 0.4—0.5 mm thick. Pericarp (or its outer layer) easily detached and ruptured, 1—2—layered, 10—40(50) μm, without papillae. Seed visually red, without keel. Testa smooth or undulate, 10—15 μm or 17—25 μm thick (both types were noted in seeds with horizontal embryos), with stalactites.

*O. macrosperma* (Hook. f.) S. Fuentes, Uotila & Borsch: 1) Falkland Islands, 1839-1843, J.D. H[ooker] *s.n.* (BM-000993193); 2) USA, California, Santa Barbara, X.1957, H.M. Pollard *s.n.* (W-21431); 3) Bolivia, Cochabamba, III.1995, N. Ritter *s.n.* (W-02936). — Fruit ovoid or obovate, 1.2—1.7 mm long, 0.5-0.8 mm thick. Pericarp easily detached from the seed and ruptured, (3—4)5—9—layered, not equal in thickness in different parts of the fruit (from (30)50 to 130 μm), without papillae. Seed visually red, without keel, 0.9—1.1 mm in diameter and ca. 0.4 mm thick. Testa smooth, 12—20 μm thick, with stalactites.

*O. rubra* (L.) S. Fuentes, Uotila & Borsch: 1) [European Russia], Tula city, without date, V.Ya. Tsinger, D.A. Kozhevnikov *613* (MW); 2) Kazakhstan, Heptapotamia, Aktogai, A. Sukhorukov & M. Lomonosova *s.n.* (MW). — (Fruits with both vertical and horizontal seed embryo were examined, but without structural differences in both seed types): Fruit 0.7—0.9 (sample 1) and 1.1—1.3 (sample 2) mm in diameter, 0.35—0.4 and 0.5—0.55 mm thick, respectively. Pericarp 1—2—layered, its outer (or single) layer easily detached and ruptured, 15—30 μm thick, without papillae (mamillate). Seed visually red, without keel. Testa 10—15 μm thick, smooth, with stalactites.

*O. urbica* (L.) S. Fuentes, Uotila & Borsch: [European Russia], Kalmykia, Chernozemelsky distr., Tavan-Gashun, X.1996, Neronov *s.n.* (MW). — Fruit 1—1.2 mm in diameter, 0.6—0.7 mm thick. Pericarp scraped off the seed, 1—2—layered, cells with dark-coloured protoplasts. Seed visually black, without keel. Testa 42—50 µm thick, with stalactites.

*Spinacia oleracea* L.: [Russia], Vologda prov., 1180, N.A. Ivanitsky *s.n.* (MW). — Fruit 2.5—3 mm in diameter, 1.2—1.3 mm thick. Pericarp 1—layered, ca. 5 μm thick, without papillae. Seed yellow-brownish, without keel. Testa ca. 15 μm, without stalactites.

*Suckleya suckleyana* Rydb.: USA, New Mexico, San Miguel co., VII.1984, Hill *14611* (GH). — Fruit ovoid, ca. 3 mm long, compressed, 0.5 mm thick. Pericarp 1—layered, ca. 5 μm thick, with a few vascular bundles, tighly adjoining the seed but scraped off, some cells with small protuberances (mammillae) on their outer walls. Seed yellow. Testa 7—10 μm thick.

*Teloxys aristata* (L.) Moq.: [European Russia], Michurinsk distr., Kochetovka-2, alien, VIII.2001, A. Sukhorukov *s.n.* (MW). — Fruit 0.7—0.8 mm in diameter, 0.4 mm thick. Pericarp scraped off the seed, 2—layered, 7—15(20) μm, without papillae. Seed blackish, keeled. Testa 12—15 μm, smooth, without stalactites.
